# Genetic manipulation of the human gut bacterium *Eggerthella lenta* reveals a widespread family of transcriptional regulators

**DOI:** 10.1038/s41467-022-33576-3

**Published:** 2022-12-09

**Authors:** Xueyang Dong, Ben G. H. Guthrie, Margaret Alexander, Cecilia Noecker, Lorenzo Ramirez, Nathaniel R. Glasser, Peter J. Turnbaugh, Emily P. Balskus

**Affiliations:** 1 Department of Chemistry and Chemical Biology, Harvard University, Cambridge, MA 02138 USA; 2 Department of Microbiology & Immunology, University of California San Francisco, San Francisco, CA 94143 USA; 3 Chan Zuckerberg Biohub, San Francisco, CA 94158 USA; 4 Howard Hughes Medical Institute, Harvard University, Cambridge, MA 02138 USA

**Keywords:** Bacterial genetics, Transcriptional regulatory elements

## Abstract

*Eggerthella lenta* is a prevalent human gut Actinobacterium implicated in drug, dietary phytochemical, and bile acid metabolism and associated with multiple human diseases. No genetic tools are currently available for the direct manipulation of *E. lenta*. Here, we construct shuttle vectors and develop methods to transform *E. lenta* and other Coriobacteriia. With these tools, we characterize endogenous *E. lenta* constitutive and inducible promoters using a reporter system and construct inducible expression systems, enabling tunable gene regulation. We also achieve genome editing by harnessing an endogenous type I-C CRISPR-Cas system. Using these tools to perform genetic knockout and complementation, we dissect the functions of regulatory proteins and enzymes involved in catechol metabolism, revealing a previously unappreciated family of membrane-spanning LuxR-type transcriptional regulators. Finally, we employ our genetic toolbox to study the effects of *E. lenta* genes on mammalian host biology. By greatly expanding our ability to study and engineer gut Coriobacteriia, these tools will reveal mechanistic details of host-microbe interactions and provide a roadmap for genetic manipulation of other understudied human gut bacteria.

## Abstract (web summary)

*Eggerthella lenta* is a prominent human gut bacterium implicated in several physiological processes, but its study has remained limited. Here, by developing a genetic toolbox for *E. lenta*, the authors provide insights into how the bacterium regulates drug and dietary compound metabolism.

## Introduction

*Eggerthella lenta* is an anaerobic Gram-positive Coriobacteriia found in the gastrointestinal tracts of around 80% of humans^1^. This gut organism has received attention because of its strong links to human health and its unique metabolic capabilities. *E. lenta* can cause bloodstream infections and is considered an opportunistic human pathogen^2,3^. *Eggerthella* species are also associated with several chronic human diseases, including asthma^4^, renal disease^5^, multiple sclerosis^6^, and rheumatoid arthritis^7^, although a causal role for *Eggerthella* in these disorders has not yet been established. *E. lenta* and related human gut Coriobacteriia also perform a wide variety of metabolic transformations, including inactivation of the widely used cardiac drug digoxin^8,9^, various reactions of dietary phytochemicals^10,11^, dehydroxylation of catechols^12,13^, and metabolism of bile acids^14,15^. Gaining a mechanistic understanding of these metabolic activities and their regulation in a host setting could better inform efforts to modulate gut microbial activities to improve human health.

The interactions of *E. lenta* and closely related *Gordonibacter* species with catechols (compounds containing a 1,2-dihydroxylated aromatic ring) highlight intriguing fundamental questions. These gut organisms use a recently discovered class of molybdenum-dependent enzymes to catalyze the chemically challenging removal of a hydroxyl group from catechol substrates^12^. This dehydroxylation reaction occurs on a wide range of catechols in the human gut, including dietary phytochemicals and host neurotransmitters, likely altering their bioactivity and bioavailability. Recently identified catechol dehydroxylases include the *E. lenta* enzymes dopamine dehydroxylase (Dadh), which acts on the catecholamine neurotransmitters dopamine and norepinephrine, hydrocaffeic acid dehydroxylase (Hcdh), and catechin dehydroxylase (Cadh), as well as the *Gordonibacter* enzymes 3,4-dihydroxyphenylacetic acid dehydroxylase (Dodh) and catechol lignan dehydroxylase (Cldh)^11–13^. These enzymes are expressed with high specificity in response to individual catechol substrates, facilitating their discovery using RNA-sequencing and activity-guided purification. However, the mechanisms underlying the regulation of these metabolic activities in *E. lenta* are completely uncharacterized.

Genetically manipulating pathways in *E. lenta*, including critical genes involved in catechol metabolism, could elucidate fundamental biological mechanisms and impact human health. However, the whole Coriobacteriia taxon, including *E. lenta*, is currently genetically intractable. Recently, genome-scale analyses of large *E. lenta* strain collections, combined with culture-based experiments, revealed antibiotic resistance phenotypes, genes predictive of intra-species competitive fitness^1^, genes involved in intestinal Th17 cell activation^16^, and CRISPR-Cas systems^17^. These studies included whole genome sequencing, comparative genomics^1^, RNA sequencing^11,12^, and animal experiments using natural *E. lenta* isolates with differing gene content^16^. However, the lack of genetic tools to directly manipulate *E. lenta* and other Coriobacteriia species has been a major barrier for elucidating mechanistic details of their genetic regulation, biochemical processes, and interactions with mammalian hosts and other microbes.

Here, we report a systematic toolkit to genetically manipulate gut Coriobacteriia. We rationally designed *E. lenta* shuttle plasmids with different antibiotic selection markers and achieved efficient transformation into an *E. lenta* type strain. We then developed a reporter system, inducible expression systems, and methods for both genome engineering and gene complementation of *E. lenta*. We used this toolkit to characterize the mechanisms underlying the regulation of catechol dehydroxylase enzyme expression. Our work uncovered the critical role of a unique class of LuxR-type transcriptional regulators, which possess an N-terminal 12-transmembrane helix domain, in activating the expression of catechol dehydroxylases and other metabolic enzymes. Finally, we used our tools to validate that the *cgr* operon, a strain-variable locus in *E. lenta* that encodes the digoxin-metabolizing enzyme, is necessary for Th17 activation in vivo by performing targeted gene deletion and in vivo experiments. The development of this genetic toolkit now sets the stage for additional efforts that will reveal mechanistic details of human gut Coriobacteriia-host interactions. Our workflow can also serve as a model for the development of similar approaches for genetic manipulation of other understudied human gut bacteria.

## Results

### Construction and transformation of shuttle plasmids for *E. lenta*

**Fig. 1:**
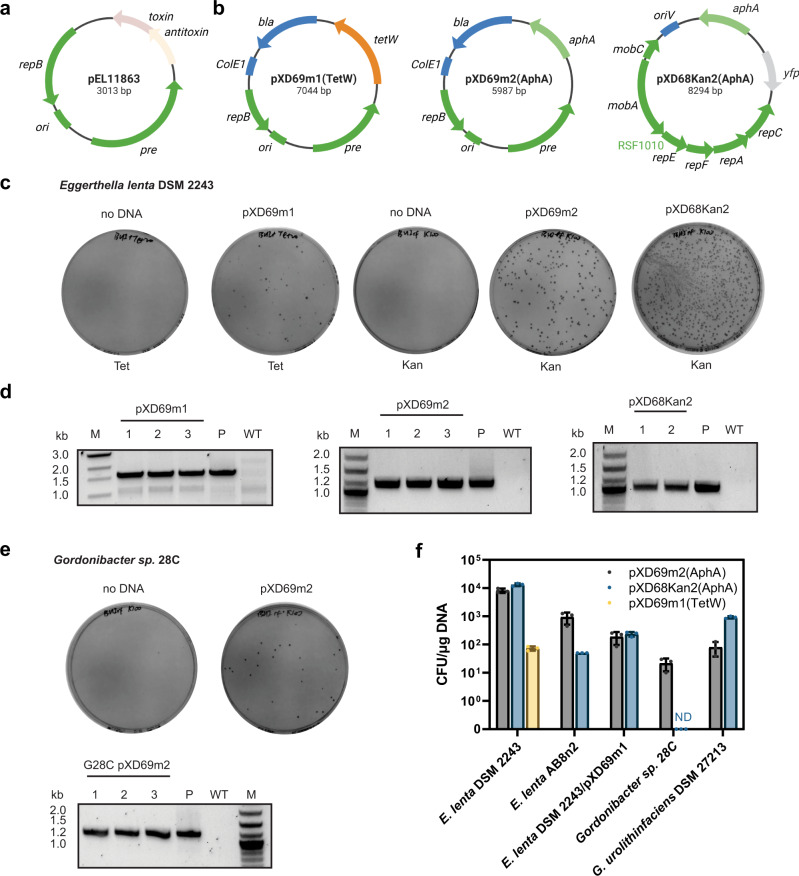
Engineering of an *E. lenta* endogenous plasmid enables efficient transformation of *E. lenta* and *Gordonibacter*. **a** An *E. lenta* endogenous plasmid pEL11863 was chosen for further engineering. **b** Construction of shuttle plasmids pXD69m1 and pXD69m2, and the broad-host-range plasmid pXD68Kan2. Shuttle plasmids pXD69m1 and pXD69m2 were constructed by assembly of *E. lenta* pEL11863 replicon (dark green), *E. coli* ColE1 origin and *bla* ampicillin-resistance gene (blue), and *E. lenta* antibiotic resistance gene *tetW* (orange) or *aphA* (light green). pXD68Kan2 was constructed from RSF1010-based broad host-range plasmid pAM5409, with antibiotic resistance gene replaced by *aphA*, and the RSF1010 replicon (dark green) and *yfp* (grey) originally on pAM5409 were retained. **c** Plasmids pXD69m1, pXD69m2 and pXD68Kan2 can be transformed into *E. lenta* DSM 2243 using electroporation. **d** Plasmid-specific PCR confirmed plasmid presence within individual colonies. Plasmids (P) and WT gDNA were used as control templates. Amplified regions are indicated in Supplementary Fig. 1a. M: DNA ladder. **e** Plasmid pXD69m2 can be transformed into *Gordonibacter sp*. 28C using electroporation. **f** The transformation efficiency of plasmids pXD69m1, pXD69m2 and pXD68Kan2 into different *E. lenta* and *Gordonibacter* strains. ND: colonies not detected. Data in panel **f** are represented as mean ± SD with *n* *=* 3 biological replicates. Source data are provided as a Source Data file.

To construct a shuttle vector between *E. coli* and *E. lenta*, we searched for potential replicative origins on previously reported endogenous *E. lenta* circular plasmids^1^. *E. lenta* DSM 11863 harbours a 3.0 kb cryptic plasmid (accession NZ_PPUC01000060.1) which we designated pEL11863 (Fig. 1a). pEL11863 contains two genes predicted to be involved in plasmid replication and maintenance: *repB*, encoding a replicative protein, and *pre*, encoding a hypothetical protein annotated as a plasmid recombination enzyme. These two genes are flanked by a 0.5 kb intergenic region containing AT-rich repeated sequences, which are indicative of the origin of replication. The rest of pEL11863 contains a polycistronic ORF that encodes a toxin-antitoxin module, which is characteristic of the bacterial plasmid addiction systems that maintain plasmid stability^18^. We reasoned that *repB*, *pre* and the intergenic region might be sufficient for plasmid replication and maintenance in *E. lenta*, and we chose pEL11863 for further engineering.

An antibiotic resistance gene is required for selection of transformants. It has been reported that predicted aminoglycoside phosphotransferase AphA confers kanamycin resistance to certain *E. lenta* strains and that putative ribosomal protection protein TetW provides tetracycline resistance^1^. Though these proteins can protect the heterologous bacterial host *E. coli* from the corresponding antibiotics^1^, their utilization as selection marker genes in *E. lenta* had not been demonstrated. We added these two antibiotic resistance genes and their native promoters to our plasmid by ligating the predicted replicon on pEL11863 with an *E. lenta* antibiotic resistance gene, the *E. coli* replicative origin and the ampicillin-resistance gene *bla* to make shuttle vectors pXD69m1(TetW) and pXD69m2(AphA), respectively (Fig. 1b).

With these two shuttle plasmids constructed, we next screened different *E. lenta* strains for their ability to be transformed using electroporation. In our initial trials, we used an aqueous solution containing 10% glycerol as an electroporation buffer to prepare electrocompetent cells. Among the strains tested, *E. lenta* DSM 2243 had the highest transformation efficiency, as it could be transformed with either shuttle plasmid (Fig. 1c). The kanamycin-resistance plasmid pXD69m2 showed higher transformation efficiency (8.1 × 10^3^ ± 1.4 × 10^3^ colony-forming units (CFUs)/µg DNA), whereas the tetracycline-resistance plasmid pXD69m1 exhibited lower efficiency (73 ± 12 CFUs/µg DNA) (Fig. 1f). Both plasmids were detected within the transformants using plasmid-specific PCR (Fig. 1d; Supplementary Fig. 1a). These transformants displayed steady growth in liquid medium supplemented with the corresponding antibiotics (Supplementary Fig. 1b), confirming *aphA* and *tetW* are effective selection markers for *E. lenta*. We further screened electroporation conditions to identify an optimal workflow (Supplementary Fig. 1c, d).

We next aimed to use our optimized electroporation protocol to facilitate the transformation of additional plasmids into *E. lenta* DSM 2243. This could enable co-transformation of expression vectors to simultaneously express multiple recombinant proteins or construct sophisticated gene regulation circuits. A previous study employed in situ bacterial conjugation to transform a plasmid containing the broad host range replicon RSF1010 into a diverse gut microbial community and obtained an *Eggerthella* transconjugant^19^. However, it was unclear which species of *Eggerthella* was conjugated and whether the RSF1010 plasmid could be introduced into *E. lenta* using our electroporation protocol. To address these questions, we constructed another *aphA*-encoding plasmid named pXD68Kan2 containing the RSF1010 replicon^20^ (Fig. 1b). We found that pXD68Kan2 can also be efficiently transformed into *E. lenta* DSM 2243 (1.3 × 10^4^ ± 1.5 × 10^3^ CFUs/µg DNA) (Fig. 1c, d, f) and the transformants also displayed steady growth in liquid medium (Supplementary Fig. 1b). To test the possibility of delivering multiple plasmids into *E. lenta* DSM 2243, we prepared competent cells of *E. lenta* DSM 2243 harbouring pXD69m1 (*E. lenta* DSM 2243/pXD69m1), and then transformed this strain with either pXD69m2, which contains the same replicon as pXD69m1, or pXD68Kan2, which contains a different replicon. Using kanamycin/tetracycline dual antibiotic selection, we found both plasmids could be transformed into *E. lenta* DSM 2243/pXD69m1 with similar transformation efficiencies (1.9 × 10^2^ ± 9 × 10^1^ CFUs/µg DNA for pXD69m2, 2.4 × 10^2^ ± 4 × 10^1^ CFUs/µg for pXD68Kan2) (Fig. 1f). This will enable further efforts to establish more complex genetic circuits for *E. lenta*. We also assessed plasmid maintenance in *E. lenta* DSM 2243 by culturing *E. lenta* strains in the absence of antibiotics, and we found that pXD69m2 and pXD68Kan2 plasmids exhibited higher segregation stability than pXD69m1 (Supplementary Fig. 1e).

We next tested if our shuttle plasmids could be delivered to closely related bacteria using our optimal electroporation conditions. We tested additional *E. lenta* strains and *Gordonibacter* species in our Coriobacteriia collection that were reported to be kanamycin-sensitive^1^. *E. lenta* AB8n2 was transformable with both pXD69m2 (9.3 × 10^2^ ± 4.2 × 10^2^ CFUs/µg DNA) and pXD68Kan2 (50 ± 0 CFUs/µg DNA) (Fig. 1f; Supplementary Fig. 1f). *Gordonibacter* sp. 28 C could be transformed with pXD69m2 (22 ± 10 CFUs/µg DNA, Fig. 1e, f) but not pXD68Kan2. *Gordonibacter urolithinfaciens* DSM 27213 was transformable with both pXD69m2 (80 ± 42 CFUs/µg DNA) and pXD68Kan2 (9.2 × 10^2^ ± 8 × 10^1^ CFUs/µg DNA) (Fig. 1f; Supplementary Fig. 1f). These results suggest that the pEL11863-derived and RSF1010-based replicons are replicative across multiple species of Coriobacteriia. Together, our shuttle vectors and transformation protocol provide a means of delivering genetic payloads to formerly genetically intractable gut Coriobacteriia.

### Development of *lacZ* as reporter gene for *E. lenta*

We next aimed to expand our toolkit by characterizing and engineering the genetic parts and methodologies needed for functional characterization efforts. Notably, we required a set of predictable genetic components to drive heterologous gene expression; however, our understanding of *E. lenta* gene regulation is extremely limited. To address this obstacle, we assessed the activity of different endogenous *E. lenta* promoters.

**Fig. 2:**
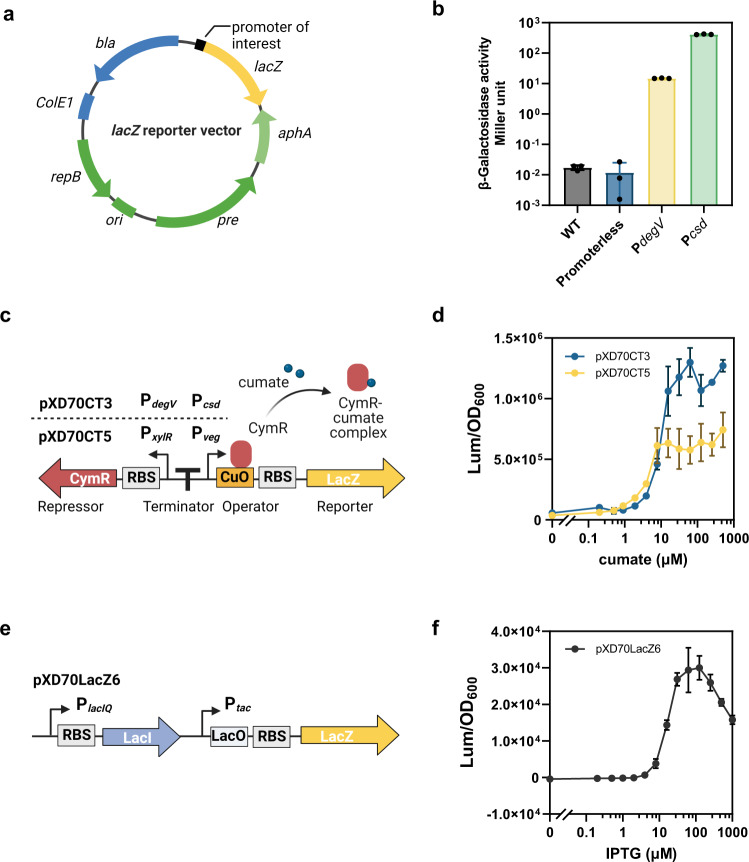
A *lacZ* reporter system enables characterization of *E. lenta* endogenous promoters and synthetic inducible expression systems. **a** *E. coli lacZ* is introduced into pXD69m2 vector as a reporter gene in *E. lenta*. **b** *LacZ* assays revealed a high dynamic range for the *lacZ* reporter system in *E. lenta*. β-Gal activity was normalized to Miller units. **c** Design of cumate-inducible expression systems using the CymR repressor and CuO, expressed under the control of *Bacillus* and *E. lenta* promoters in plasmids pXD70CT5 and pXD70CT3, respectively. **d** *LacZ* assays revealed tight regulation of cumate-inducible expression systems pXD70CT3 and pXD70CT5. **e** Design of IPTG-inducible expression system in plasmid pXD70LacZ6. **f** *LacZ* assays for pXD70LacZ6. Data in panels **b**, **d**, and **f** are represented as mean ± SD with *n* = 3 biological replicates. Source data are provided as a Source Data file.

Reporter systems are frequently used for evaluation of promoter strength and gene expression levels under different conditions. To develop such a system for *E. lenta*, we first tested if β-galactosidase (β-Gal) could be used as a reporter gene. No background β-Gal activity was detected in the *E. lenta* DSM 2243 wild-type (WT) strain (Fig. 2b), confirming that it lacks endogenous β-Gal enzymes. We then cloned *E. coli lacZ* encoding β-Gal into the pXD69m2 vector (Fig. 2a). We placed *lacZ* downstream of two different *E. lenta* native promoters that RNA-seq experiments indicate are expressed constitutively at high levels^13^. The first native constitutive promoter P_*degV*_ controls the expression of Elen_1941, a gene encoding a conserved degV family protein. Another stronger constitutive promoter P_*csd*_, identified from previous RNA-seq data^13^ controls the expression of a predicted cold-shock DNA-binding domain protein Elen_0979. A promoterless *lacZ* fusion was also constructed as a negative control. We transformed these reporter plasmids into *E. lenta* DSM 2243 and found the *lacZ* reporters showed a high dynamic range of over four orders of magnitude in *E. lenta* (Fig. 2b). The P_*csd*_ and P_*degV*_ constructs both exhibited robust β-Gal activity consistent with the expression levels observed in earlier RNA-seq experiments (Fig. 2b)^13^. Finally, we further optimized experimental conditions to increase the throughput of the β-Gal assays (Supplementary Fig. 2a), establishing the *lacZ* reporter as a convenient tool to measure promoter activity in *E. lenta*.

### Construction and characterization of synthetic inducible expression systems

Development of an inducible expression system independent of host signalling pathways would allow temporal control of gene expression in *E. lenta*. Such a system could better support real-time analysis of the functions of microbiome gene products^21^ and optimize the balance between cell fitness and product synthesis for metabolic engineering^22^. To identify an optimal inducible expression system for *E. lenta*, we first attempted to adapt several systems developed for other bacteria. We observed tight regulation of expression and strong induction with a cumate-inducible system^23^. In this construct, pXD70CT5, we introduced cumate-regulatory elements, including a CymR repressor and a cumate operator (CuO) from a *Bacillus* vector^23^ into our *lacZ* vector (Fig. 2c). In the absence of cumate, we observed low expression levels of *lacZ*. At cumate concentrations below 8 μM, *lacZ* expression increased in a dose-dependent manner (Fig. 2d). At cumate concentrations above 8 μM, *lacZ* expression was fully derepressed, with a 20-fold change between fully induced and uninduced cultures (Fig. 2d). Cumate had no significant influence on bacterial growth at concentrations as high as 500 μM (Supplementary Fig. 2b).

Inspired by the modular design of this cumate-inducible expression system, we next attempted to build similar genetic expression systems using *E. lenta* endogenous promoters. In our previous experiments, the activity of P_*degV*_ and P_*csd*_ varied by over 25-fold. In construct pXD70CT3, we used P_*degV*_ to control expression of repressor CymR, and used P_*csd*_ inserted with a CuO to drive *lacZ* expression (Fig. 2c; Supplementary Fig. 2c). This system also showed tight gene regulation, exhibiting a 22-fold change in *lacZ* expression levels between fully induced and uninduced cultures, similar to pXD70CT5 (Fig. 2d). Full de-repression of *lacZ* was reached at a cumate concentration of 62.5 μM using pXD70CT3 (Fig. 2d), which is significantly higher than that achieved with pXD70CT5 and is likely due to increased repressor expression under promoter P_*degV*_. Thus, two tightly controlled and highly inducible expression systems, made from either *Bacillus* or endogenous *E. lenta* promoters, have been developed for *E. lenta*.

In addition to the cumate-inducible systems, we found that *E. lenta* harbouring the pXD70LacZ6 plasmid, which features a LacI repressor/LacO operator construct (Fig. 2e) adapted from *E. coli* vector pMAL-c2x^24^, could respond to IPTG (isopropyl β-D-1-thiogalactopyranoside). This construct exhibited virtually no detectable *lacZ* activity at IPTG concentrations below 2 μM and reached maximum *lacZ* expression levels at an IPTG concentration of 62.5–125 μM (Fig. 2f; Supplementary Fig. 2d). The maximum *lacZ* levels of pXD70LacZ6 were significantly lower than those in cumate-inducible expression systems, indicating that this construct might be useful for expressing genes at low levels. Replacing the P_*tac*_ promoter that controls the *lacZ* expression with stronger constitutive promoters could potentially yield IPTG-inducible constructs with broader dynamic ranges in *E. lenta*.

### Characterization of native inducible expression systems in *E. lenta*

**Fig. 3:**
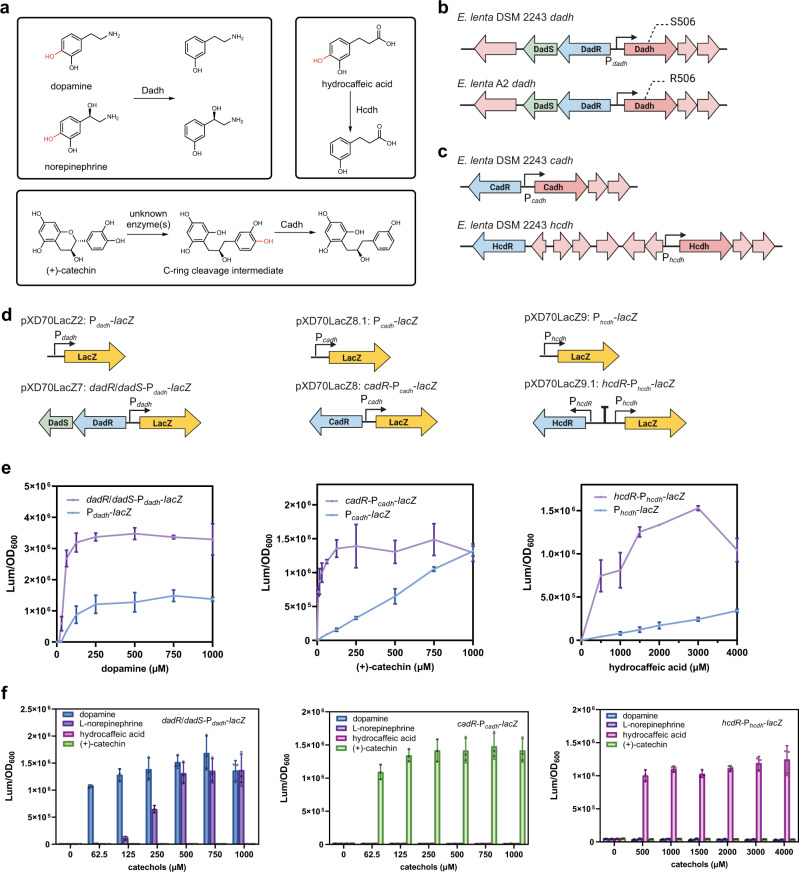
Promoters of *E. lenta* catechol dehydroxylases exhibit high specificity for individual enzyme substrates. **a** Key reactions catalyzed by *E. lenta* catechol dehydroxylases. **b** Genomic contexts of *dadh* in the non-dopamine metabolizing strain *E. lenta* DSM 2243 and the dopamine-metabolizing *E. lenta* A2. **c** Genomic contexts of *cadh* and *hcdh* in *E. lenta* DSM 2243. **d** Design of P_*dadh*_ reporters pXD70LacZ2 and pXD70LacZ7, P_*cadh*_ reporters pXD70LacZ8.1 and pXD70LacZ8, and P_*hcdh*_ reporters pXD70LacZ9 and pXD70LacZ9.1. **e** *LacZ* assays revealed the transcriptional response of catechol dehydroxylase promoter reporters to corresponding catechols. **f** *LacZ* assays revealed the high specificity of each catechol dehydroxylase promoter construct for individual enzyme substrates. Data represented as mean ± SD with *n* = 3 biological replicates for **e** and **f**. Source data are provided as a Source Data file.

Native bacterial promoters that respond to host-derived and dietary compounds have been engineered to regulate gene expression in a variety of human gut bacteria^25–27^. As a formerly intractable species, the repertoire of native inducible promoters in *E. lenta* is largely unknown. As highlighted earlier, the expression of various *E. lenta* catechol dehydroxylase enzymes is specifically upregulated by their substrates^12,13^ (Fig. 3a), indicating the presence of catechol-inducible promoters regulated by uncharacterized transcriptional factors.

We thus harnessed our *lacZ* reporter system to identify and characterize the *cis*-acting elements that control the transcriptional response of *E. lenta* to dopamine. Notably, there is considerable strain-level variability in dopamine dehydroxylation among *E. lenta*. Sequenced *E. lenta* strains all encode Dadh, but only some metabolize dopamine^13^. This difference in activity strongly correlates with a single nucleotide polymorphism in *dadh* that introduces an amino acid variation, with metabolizing strains encoding an arginine at position 506 of Dadh, and strains with little or no metabolism possessing a serine (Fig. 3b)^13^. Though *E. lenta* DSM 2243 does not metabolize dopamine, the inactive variant of *dadh* is still upregulated by dopamine^13^, which suggests the elements regulating *dadh* expression are still functional. Importantly, the *dadh* gene cluster of *E. lenta* DSM 2243 closely resembles that of the dopamine-metabolizing strain *E. lenta* A2 (Fig. 3b). These features make *E. lenta* DSM 2243 an amenable host to study the transcriptional response to dopamine.

We first tested if the *dadh* promoter reporter P_*dadh*_-*lacZ* (pXD70LacZ2) could be induced by dopamine (Fig. 3d). Consistent with the hypothesis that P_*dadh*_ controls the expression of *dadh* in response to dopamine, *E. lenta* DSM 2243 harbouring the pXD70LacZ2 construct responded to dopamine in a concentration-dependent manner, reaching maximum activity at 250 μM (Fig. 3e). Since we did not include any *trans*-acting factors in pXD70LacZ2, its induction must be mediated by transcriptional regulators encoded in the chromosome. We hypothesized that regulation of *dadh* in response to dopamine might be mediated by locus-specific transcriptional regulators, which prompted us to examine the genomic neighbourhoods of this gene. The genomic context of *dadh* includes two putative transcriptional regulators encoded in the opposite orientation, one annotated as a LuxR-type transcriptional regulator (Elen_0473) and the other a LysR-type transcriptional regulator (Elen_0474) (Fig. 3b). We designate these putative regulators as DadR (*dadh* LuxR-type regulator) and DadS (*dadh* LysR-type regulator), respectively. Interestingly, in addition to a C-terminal helix-turn-helix (HTH) DNA-binding domain (DBD), which is a typical feature of LuxR-type regulators^28^, DadR possesses a predicted N-terminal 12-transmembrane helix (TM) domain (Supplementary Fig. 3a). This unusual domain architecture has not previously been reported for this regulator family. Notably, predicted 12-TM LuxRs are located near genes encoding multiple other important *E. lenta* metabolic enzymes, including the catechol dehydroxylases Cadh and Hcdh^12^ (Fig. 3c), and the enzymes Cgr2^8,9^ and Ber^11^ that metabolize the cardiac glycoside digoxin and the plant lignan pinoresinol, respectively (Supplementary Fig. 3b). The domain architectures and genomic contexts of these LuxRs led us to propose they might mediate expression of co-localized metabolic enzymes.

We then sought to characterize the biological functions of these 12-TM LuxRs in *E. lenta*, beginning with the putative dopamine metabolism regulator DadR. To address the influence of DadR and DadS on the expression of genes downstream of P_*dadh*_, we constructed a *dadR/dadS*-P_*dadh*_-*lacZ* fusion (pXD70LacZ7, Fig. 3d). pXD70LacZ7 showed a significantly stronger response to dopamine compared to pXD70LacZ2 (Fig. 3e). We reasoned that in pXD70LacZ7, DadR and DadS are likely overexpressed compared to the WT strain harbouring pXD70LacZ2 as the pXD70LacZ7 plasmid supplies additional copies of these genes, ultimately resulting in higher expression of *lacZ*. These results indicated that DadR and/or DadS likely mediate activation of P_*dadh*_.

Because the induction of catechol dehydroxylases is specific to their individual substrates^12^, we next tested whether the response of the *lacZ* reporters reflects the substrate specificity of Dadh. We observed that the response of pXD70LacZ7 is indeed specific to Dadh substrates (Fig. 3f), with expression strongly induced by dopamine and norepinephrine, but not by hydrocaffeic acid or (+)-catechin, which are substrate or substrate precursor of other catechol dehydroxylases and are not accepted by Dadh in vitro^12^.

We then sought to characterize the specificities of additional catechol dehydroxylase promoters towards their inducers. *E. lenta* DSM 2243 metabolizes (+)-catechin by first cleaving its C-ring using uncharacterized enzymes followed by B-ring dehydroxylation using Cadh (Fig. 3a)^12^. It also dehydroxylates hydrocaffeic acid using Hcdh (Fig. 3a)^12^. 12-TM LuxR-type regulators, which we designate as CadR (*cadh* regulator) and HcdR (*hcdh* regulator), are encoded close to *cadh* and *hcdh* (Fig. 3c). Unlike *dadR*, no LysR-type regulators are encoded next to these LuxRs. We hypothesized that CadR and HcdR regulate expression of *cadh* and *hcdh*, respectively, in response to their specific substrates. To assess the promoter responses towards different catechols, we constructed *cadh* promoter reporters with regulator-encoding gene *cadR*, pXD70LacZ8 (*cadR*-P_*cadh*_-*lacZ*), and without *cadR*, pXD70LacZ8.1 (P_*cadh*_-*lacZ*), respectively (Fig. 3d). For the *hcdh* promoter reporters, we constructed pXD70LacZ9 (P_*hcdh*_-*lacZ*) and pXD70LacZ9.1 (*hcdR*-P_hcdh_-*lacZ*) which contains the regulator-encoding gene *hcdR* under its native promoter segregated from the reporter sequences by a transcriptional terminator (Fig. 3d). Both pXD70LacZ8 and pXD70LacZ8.1 responded to (+)-catechin, the precursor of the Cadh substrate, and both pXD70LacZ9 and pXD70LacZ9.1 responded to hydrocaffeic acid (Fig. 3e). Interestingly, constructs that included the putative regulator-encoding gene (pXD70LacZ8 for P_*cadh*_ and pXD70LacZ9.1 for P_*hcdh*_) exhibited stronger *lacZ* induction than the corresponding constructs lacking these regulators (Fig. 3e), suggesting CadR and HcdR might also be transcriptional activators. Congruent to the observed specificity of P_*dadh*_, pXD70LacZ8 only responded to (+)-catechin, and pXD70LacZ9.1 only responded to hydrocaffeic acid (Fig. 3f). As we could not test the actual, ring-cleaved Cadh substrate in these experiments, we cannot tell if CadR is responding to (+)-catechin or this pathway intermediate. Overall, these findings identify a panel of highly specific native *E. lenta* inducible promoters which may be further engineered and employed for regulating gene expression.

### Expression of catechol dehydroxylases in *E. lenta*

**Fig. 4:**
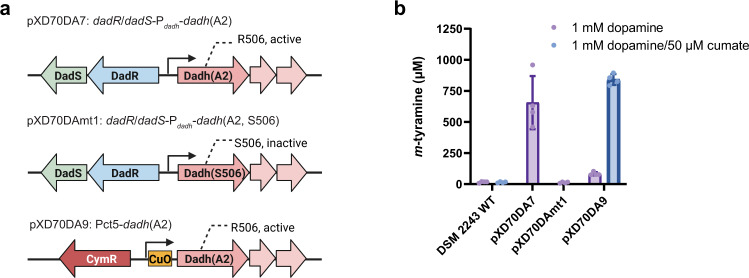
Introducing the active Dadh variant into the non-dopamine metabolizing strain *E. lenta* DSM 2243 results in dopamine dehydroxylation. **a** Schematic of pXD70DA7, pXD70DAmt1, and pXD70DA9 constructs. **b** LC-MS/MS to quantify the production of dopamine dehydroxylation metabolite *m*-tyramine after incubation with corresponding *E. lenta* cultures for 48 h. Data represented as mean ± SD with *n* = 4 biological replicates. Source data are provided as a Source Data file.

The ability to express proteins of interest in *E. lenta* could facilitate the functional and biochemical characterization of proteins that are challenging to obtain from other heterologous hosts. Heterologous expression of *E. lenta* catechol dehydroxylases has been unsuccessful to date, likely due to their complex requirements for metallocofactor assembly^12,13^. We tested our inducible systems by expressing Dadh from the dopamine metabolizing strain *E. lenta* A2 in the non-metabolizer *E. lenta* DSM 2243. We used both the native promoter P_*dadh*_ and the cumate-inducible promoter to drive expression of the active Dadh catalytic subunit (R506 variant) and two additional subunits that are encoded next to the catalytic subunit and are proposed to serve as electron transfer and membrane anchor partners (Dadh(A2)) (Fig. 4a). In construct pXD70DA7, we placed *dadR/dadS*-P_*dadh*_ upstream of *dadh*(A2) (Fig. 4a). This resembles the genomic context of *dadh* and could potentially increase its expression levels analogous to what we observed in the *lacZ* reporter assay. In construct pXD70DAmt1, we mutated the R506 in Dadh(A2) catalytic subunit on pXD70DA7 into S506 to test the influence of this substitution on dopamine metabolism (Fig. 4a). In construct pXD70DA9, we used the same cumate-inducible promoter as in pXD70CT5 (Pct5) to drive expression of Dadh(A2) (Fig. 4a).

Testing these Dadh expression constructs revealed successful implementation of this strategy. Incubating *E. lenta* DSM 2243 cultures harbouring pXD70DA7 with 1 mM dopamine for 48 h resulted in steady dopamine metabolism, in contrast to the WT strain and cultures harbouring pXD70mt1 which showed <2% conversion (Fig. 4b). *E. lenta* DSM 2243 cultures harbouring pXD70DA9 were incubated with 1 mM dopamine with or without 50 μM cumate for 48 h. The cultures with cumate induction showed high activity, metabolizing ~84% of the dopamine, while the cultures lacking cumate induction showed lower conversion of dopamine (~8%), and cumate didn’t induce any activity in the WT strain (Fig. 4b). These results show that introducing the active Dadh variant (R506) into *E. lenta* DSM 2243 renders this non-metabolizing strain capable of dopamine dehydroxylation, validating this amino acid as the key determinant for Dadh activity. We also successfully demonstrated the use of a synthetic inducible promoter for regulation of an *E. lenta* metabolic activity.

### Activation of a native CRISPR-Cas3 system enables markerless genomic engineering and deletion of *dadR* in *E. lenta*

**Fig. 5:**
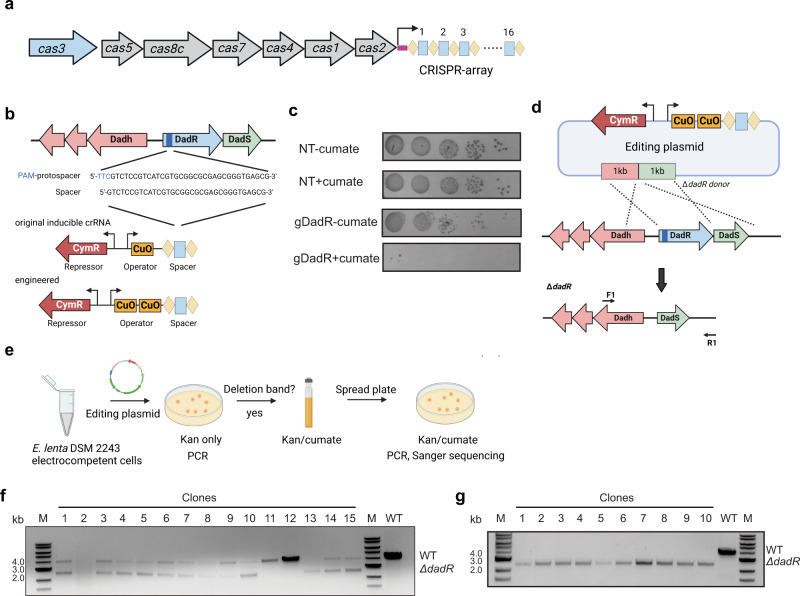
Repurposing the endogenous type I-C CRISPR-Cas system in *E. lenta* enables markerless deletion of dadR. **a** Architecture of the CRISPR-Cas locus in *E. lenta* DSM 2243. **b** Spacer choice for *dadR*-targeting crRNA and optimization of cumate-inducible crRNA construct. **c** Induction of *dadR*-targeting crRNA (gDadR) decreased the CFUs when compared to an uninduced control. No significant difference in CFUs was observed when the NT was induced. A single representative image is shown with *n* = 3 biological replicates. **d** Repair template is introduced to make editing plasmid pXD71Cas10.1RT. To probe potential *dadR* deletion, primers flanking the repair template were used. **e** Workflow for generating the Δ*dadR* deletion strain. **f** PCR screening of colonies formed after initial pXD71Cas10.1RT transformation revealed most colonies contained both non-edited (4 kb) and edited cells (2.5 kb). **g** PCR screening of colonies formed after spreading initial pXD71Cas10.1RT transformant cultures onto a cumate-containing plate revealed clean Δ*dadR* mutants (2.5 kb), which was further confirmed by Sanger sequencing. Experiments shown in panels **f** and **g** were performed once on randomly selected colonies. Source data are provided as a Source Data file.

Since the *E. lenta* DSM 2243 chromosome already contains the transmembrane LuxR-encoding genes, we could not draw definitive conclusions about their functions in regulating catechol dehydroxylases based on reporter assays alone. Further functional characterization of this unusual family of transcriptional regulators requires methods for targeted gene deletion in *E. lenta*. Development of gene editing systems accelerates elucidation of the functions of bacterial gene products, and CRISPR-Cas systems have been engineered as a programmable platform for gene editing in numerous bacterial species^29–32^. Several *E. lenta* isolates contain a type I-C CRISPR-Cas system which is actively transcribed and capable of targeting chromosomal DNA in the heterologous host *Pseudomonas aeruginosa* (Fig. 5a)^17^. We thus explored the possibility of repurposing the *E. lenta* CRISPR-Cas system for endogenous gene editing and applied this system to characterize the functions of the unusual 12-TM LuxR-type regulators.

To achieve self-targeting, we sought to construct a minimal CRISPR array (crRNA) to direct the Cas effectors to the host chromosome. As self-targeting typically results in strong cytotoxicity, which impairs plasmid transformation^29,30^, we used the inducible system Pct5 employed in pXD70CT5 to make a cumate-inducible *dadR*-targeting crRNA plasmid (Fig. 5b). We attempted to introduce this plasmid into *E. lenta* DSM 2243 by electroporation but observed no colonies after several attempts, likely due to cytotoxicity triggered by basal expression of self-targeting crRNA. To strengthen repression, we introduced an additional CymR repressor binding site (CuO) downstream of the existing CuO region to construct a new *dadR*-targeting plasmid pXD71Cas10.1 (Fig. 5b), which enabled formation of transformants. This new promoter displayed lower basal expression levels than the original Pct5, confirmed by β-Gal activity assays (Supplementary Fig. 4a). To test the functionality of the CRISPR-Cas system in its native host, starter cultures grown in the absence of cumate were plated on media supplemented with the selecting antibiotic and containing or lacking 50 μM cumate. We observed a 10^3^-fold decrease in CFUs when the *dadR*-targeting crRNA (gDadR) was induced, whereas no significance difference was observed when a non-targeting (NT) control crRNA was induced (Fig. 5c). This drastic cytotoxicity suggested the CRISPR-Cas system in *E. lenta* is operating with high levels of activity and target specificity.

Further PCR screening of the surviving colonies from gDadR induction showed no deletion at the target site (Supplementary Fig. 4b). Instead, we observed spacer excision via homologous recombination between the direct repeats from the pXD71Cas10.1 plasmid of the surviving colonies (Supplementary Fig. 4c). Since gene deletion requires DNA repair after self-targeting, these results suggested the *E. lenta* DNA repair process was not efficient in the absence of a repair template. We next introduced a repair template into the crRNA plasmid. The template was designed to contain sequences 1 kb upstream and 1 kb downstream of *dadR* and was inserted into pXD71Cas10.1 to generate editing plasmid pXD71Cas10.1RT (Fig. 5d). After transformation with pXD71Cas10.1RT (Fig. 5e), PCR of the transformants revealed that the majority contained a mixture of cells lacking a deletion (4 kb) and cells containing the targeted deletion (2.5 kb) (Fig. 5f). This result suggested that basal expression of crRNA and subsequent genomic editing had occurred to some extent when initial colonies formed in the absence of inducer. When these partially edited colonies were transferred to a plate containing 50 μM cumate (Fig. 5e), cells lacking the deletion were eliminated and only colonies with the targeted deletion were identified (Fig. 5g). We designated this strain as Δ*dadR*. Thus, we demonstrated the endogenous type I-C CRISPR-Cas system can be repurposed to perform genomic engineering in *E. lenta*. Since type I-C CRISPR-Cas systems exist in many other Coriobacteriia (Supplementary Fig. 4d), this approach may enable genomic engineering of other Coriobacteriia.

### 12-TM LuxRs activate catechol dehydroxylase expression in response to specific substrates

**Fig. 6:**
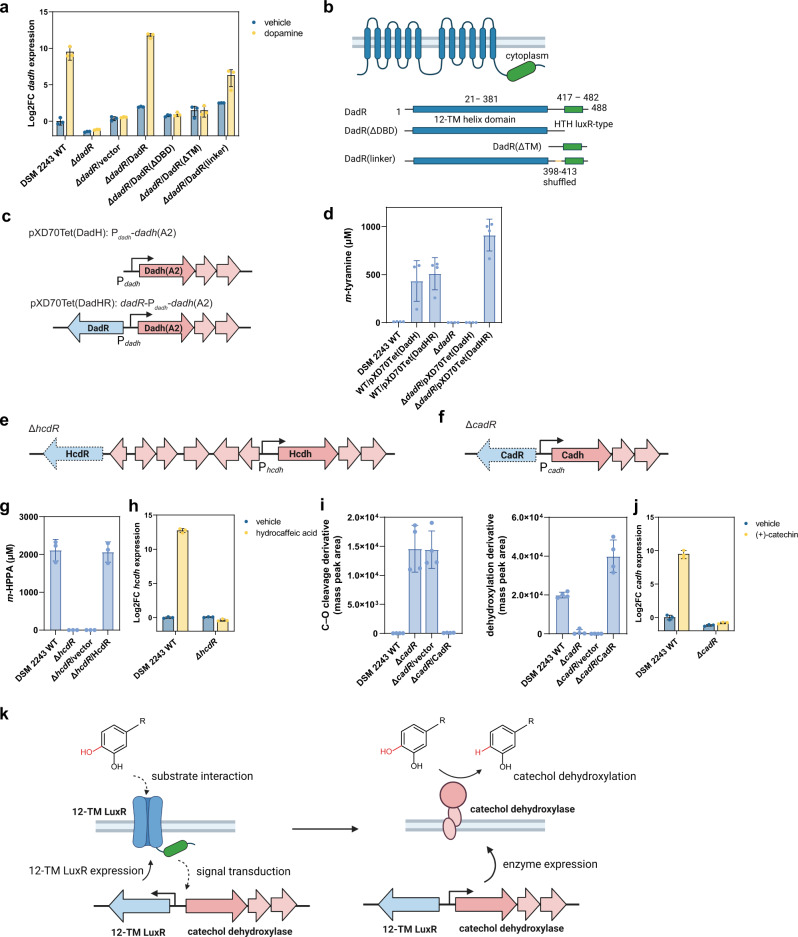
Gene deletion and complementation reveal that *E. lenta* DadR, HcdR, and CadR are transmembrane transcriptional activators. **a** RT-qPCR to test *dadh* upregulation on exposure to dopamine in *E. lenta* DSM 2243 WT, Δ*dadR* and complementation strains. **b** Schematic of the domain organization of DadR and DadR mutants examined in this work. **c** Schematic of pXD70Tet(DadH) and pXD70Tet(DadHR) constructs for testing the relevance of DadR for *E. lenta* dopamine metabolism. **d** LC-MS/MS to quantify the production of dopamine dehydroxylation metabolite *m*-tyramine after incubation with corresponding *E. lenta* cultures for 72 h. **e** Deletion of *hcdR* in *E. lenta* DSM 2243 to generate Δ*hcdR* strain. **f** Deletion of *cadR* in *E. lenta* DSM 2243 to generate Δ*cadR* strain. **g** LC-MS/MS to quantify the production of hydrocaffeic acid dehydroxylation metabolite *m*-HPPA after incubation with corresponding *E. lenta* cultures for 48 h. **h** RT-qPCR to test *hcdh* upregulation on exposure to hydrocaffeic acid in *E. lenta* DSM 2243 WT and Δ*hcdR*. **i**, LC-MS/MS to quantify the production of (+)-catechin C–O cleavage metabolite and dehydroxylation metabolite after incubation with corresponding *E. lenta* cultures for 48 h. **j** RT-qPCR to test *cadh* upregulation on exposure to (+)-catechin in *E. lenta* DSM 2243 WT and Δ*cadR*. **k** Proposed mechanisms of 12-TM LuxR-mediated gene regulation in response to catechol inducer. Data represented as mean ± S.E.M. with n = 3 biological replicates in **a**, **h**, and **j**. Data represented as mean ± SD with *n* = 4 biological replicates in **d** and **i**. Data represented as mean ± SD with n = 3 biological replicates in **g**. Source data are provided as a Source Data file.

To characterize the function of this 12-TM LuxR family, we initially tested the effects of *dadR* deletion on *dadh* expression. We first performed RT-qPCR to quantify levels of *dadh* mRNA in DSM 2243 WT and Δ*dadR* strains upon exposure to dopamine. In the presence of dopamine, *dadh* was induced to about 2^9^-fold in WT strain, whereas in Δ*dadR* strains, the levels of *dadh* expression were low and unaffected by the presence of dopamine (Fig. 6a). These data suggest DadR is responsible for activating the expression of *dadh* in the presence of dopamine.

To validate the function of DadR, we sought to complement the Δ*dadR* strain, which should restore the transcriptional response of *dadh* to dopamine. We first attempted to cure the crRNA plasmid within the Δ*dadR* strain to enable delivery of a second kanamycin-resistance plasmid. However, we found the crRNA plasmid exhibited high segregation stability, as the plated colonies were still resistant to kanamycin after five rounds of passaging in medium lacking kanamycin. This prompted us to transform the Δ*dadR* strain with a second plasmid possessing a different antibiotic resistance gene. To construct this plasmid, we cloned *dadR* under its native promoter into the pXD69m1 vector to generate tetracycline-resistance plasmid pXD70Tet(DadR). We then transformed the Δ*dadR* strain with pXD70Tet(DadR) or pXD69m1 vector (Supplementary Fig. 5a, b). RT-qPCR analysis revealed that, in the complementation strain Δ*dadR*/DadR, *dadh* basal expression levels in the absence of dopamine were about 3 times higher than in the uninduced WT, which might result from higher levels of DadR overexpressed from the plasmid. In the presence of dopamine, *dadh* was induced to about 3400 times higher than in the uninduced WT (Fig. 6a). Introduction of the empty vector (Δ*dadR*/vector) did not restore *dadh* induction (Fig. 6a). These results thus demonstrate that DadR is a bona fide LuxR-type transcriptional regulator that responds to dopamine and activates the expression of *dadh*.

We then sought to test whether *dadh* gene regulation by DadR is relevant to dopamine metabolism. *E. lenta* DSM 2243 is a dopamine non-metabolizing strain, and as shown above, introduction of an active Dadh(A2) variant to *E. lenta* DSM 2243 allows *E. lenta* DSM 2243 to metabolize dopamine. This system enabled us to test the relevance of DadR to dopamine metabolism in *E. lenta* DSM 2243. We constructed a tetracycline-resistance plasmid pXD70Tet(DadH) encoding the active Dadh variant Dadh(A2) under native P_*dadh*_ promoter without cognate regulators (Fig. 6c). We introduced this plasmid into Δ*dadR* mutant and DSM 2243 WT *E. lenta* strains. We found this plasmid could render the DSM 2243 WT strain, but not the Δ*dadR* mutant, active for dopamine dehydroxylation (Fig. 6d). This finding suggests that the lack of DadR-mediated *dadh* induction in Δ*dadR* prevents dopamine metabolism. To further confirm DadR’s role, we included *dadR* in the P_*dadh*_-Dadh(A2) construct to make another tetracycline-resistance plasmid pXD70Tet(DadHR) (Fig. 6c). This new plasmid rendered both DSM 2243 WT and Δ*dadR* mutant *E. lenta* capable of dehydroxylating dopamine (Fig. 6d). These results proved that the activation of *dadh* by DadR is essential for dopamine metabolism. To our knowledge, *E. lenta* DadR is the first functionally characterized transcriptional regulator that possess a 12-TM domain at its N-terminus, highlighting a potentially distinct mechanism of bacterial transcriptional regulation that responds to environmental cues.

To evaluate the importance of the TM domain and DBD of DadR for regulation of dopamine metabolism, we complemented the Δ*dadR* strain with plasmids encoding DadR mutants. These mutants lacked either the DBD (DadR(ΔDBD)) or 12-TM domain (DadR(ΔTM)), or contained a shuffled linker region connecting these domains (398–413) (DadR(linker)) (Fig. 6b; Supplementary Fig. 5c, d). RT-qPCR revealed no restoration of dopamine-induced *dadh* expression for Δ*dadR* strains complemented with DadR(ΔDBD) or DadR(ΔTM). In these two strains, *dadh* levels were close to the uninduced WT strain and were unaffected by dopamine (Fig. 6a). These results demonstrate that the 12-TM domain and the DBD are both essential for DadR function. Surprisingly, the strain complemented with DadR(linker) restored dopamine-induced *dadh* expression, although to a lesser extent than WT DadR. In the presence of dopamine, *dadh* expression levels in DadR(linker) reached to about 80-fold of those in the uninduced WT, which indicated the linker region might accommodate amino acid variation to some extent. In addition, to measure the expression of DadR and DadR mutants in *E. lenta*, we transformed the Δ*dadR* strain with plasmids encoding N-terminal FLAG-tagged DadR and DadR mutants. Using anti-FLAG antibody for Western blot (Supplementary Fig. 5e), we found that FLAG-DadR increased expression after exposure to dopamine, suggesting a potential autoregulation of DadR expression. Among the constructs incapable of inducing *dadh* in the presence of dopamine, the expression of FLAG-DadR(ΔTM) was confirmed by Western blot, confirming the lack of *dadh* induction in Δ*dadR*/DadR(ΔTM) was due to the inherent inactivity of this truncation mutant instead of protein degradation. Since non-specific signals prevented a clear readout of FLAG-DadR(ΔDBD) expression, we cannot exclude the possibility that insufficient expression or protein degradation of DadR(ΔDBD) leads to its observed inability to regulate *dadh* expression. These results set the stage for future site-directed mutagenesis of DadR to dissect the essential residues of the regulator.

We then sought to characterize the function of additional regulators with similar domain architecture. Though the similar domain organizations of HcdR and CadR relative to DadR imply a related function, their genomic contexts relative to their co-localized catechol dehydroxylase-encoding genes differ from that of *dadR*. Specifically, there are no LysR-type regulator-encoding genes downstream of *hcdR* and *cadR*, and *hcdR* is not encoded in direct proximity to *hcdh*. To further characterize these putative 12-TM LuxR family members, we tested whether HcdR and CadR also regulate catechol dehydroxylase expression. Using our genomic engineering method, we generated Δ*hcdR* and Δ*cadR* mutants from *E. lenta* DSM 2243 (Fig. 6e, f; Supplementary Fig. 6a, b). We found that *hcdR* deletion completely abolished hydrocaffeic acid metabolism in *E. lenta* (Fig. 6g), which is likely due to the lack of *hcdh* induction in the Δ*hcdR* mutant (Fig. 6h). Hydrocaffeic acid metabolism was restored by complementation with a *hcdR*-encoding plasmid, but not empty vector (Fig. 6g). These experiments demonstrate that HcdR regulates the transcription of *hcdh* in response to hydrocaffeic acid and is indispensable for hydrocaffeic acid metabolism. Similarly, *cadR* deletion abolished dehydroxylation, as the two-step metabolism of (+)-catechin stopped after the initial C–O bond cleavage (Fig. 6i). RT-qPCR revealed that *cadh* induction upon exposure to (+)-catechin was also abolished in *E. lenta* Δ*cadR* (Fig. 6j). The second dehydroxylation step in this pathway was restored by complementation of the Δ*cadR* mutant with *cadR* (Fig. 6i). These results show that CadR regulates the expression of the co-localized dehydroxylase *cadh* and dehydroxylation activity, without affecting the upstream C–O bond cleavage reaction. No cross-regulation was observed for HcdR and CadR, as the Δ*hcdR* mutant could completely metabolize (+)-catechin and the Δ*cadR* mutant metabolized hydrocaffeic acid (Supplementary Fig. 6c). In total, we characterized the functions of three 12-transmembrane helix LuxRs in regulating *E. lenta* enzyme expression and metabolic activity, indicating a shared role for the broader family in regulating individual metabolic activities.

### 10–12-transmembrane helix LuxRs are prevalent in Coriobacteriia

Having confirmed the roles of DadR, HcdR and CadR in regulating catechol metabolism, we assessed the diversity of related TM LuxRs in *E. lenta*. We first queried the *E. lenta* DSM 2243 genome for other proteins containing LuxR-type HTH DBDs (pfam00196) and analyzed the presence and number of TM helices in hits using the IMG/M database^33^. A total of 83 transmembrane LuxR-type regulators were found in this strain, with the majority having 10–12 N-terminal TM helices (Supplementary Data 1). These 10–12-TM LuxRs include 60 proteins possessing 12 TM helices, 8 with 11 TM helices, and 6 with 10 TM helices. These proteins share significant homology between their C-terminal DBDs (Supplementary Fig. 7a), whereas their N-terminal TM domains generally have low sequence identity (<20% amino acid identity) (Supplementary Data 2). In *E. lenta* DSM 2243, 19 TM LuxRs are encoded close to molybdenum-dependent enzymes and 50 are encoded in proximity to flavin-dependent enzymes (Supplementary Fig. 7b, c), suggesting they regulate *E. lenta* metabolism in response to many different substrates. Due to their similar domain architectures and TM helices, we consider these 10–12-TM helix LuxRs to be a previously unappreciated family of bacterial transcriptional regulators.

**Fig. 7:**
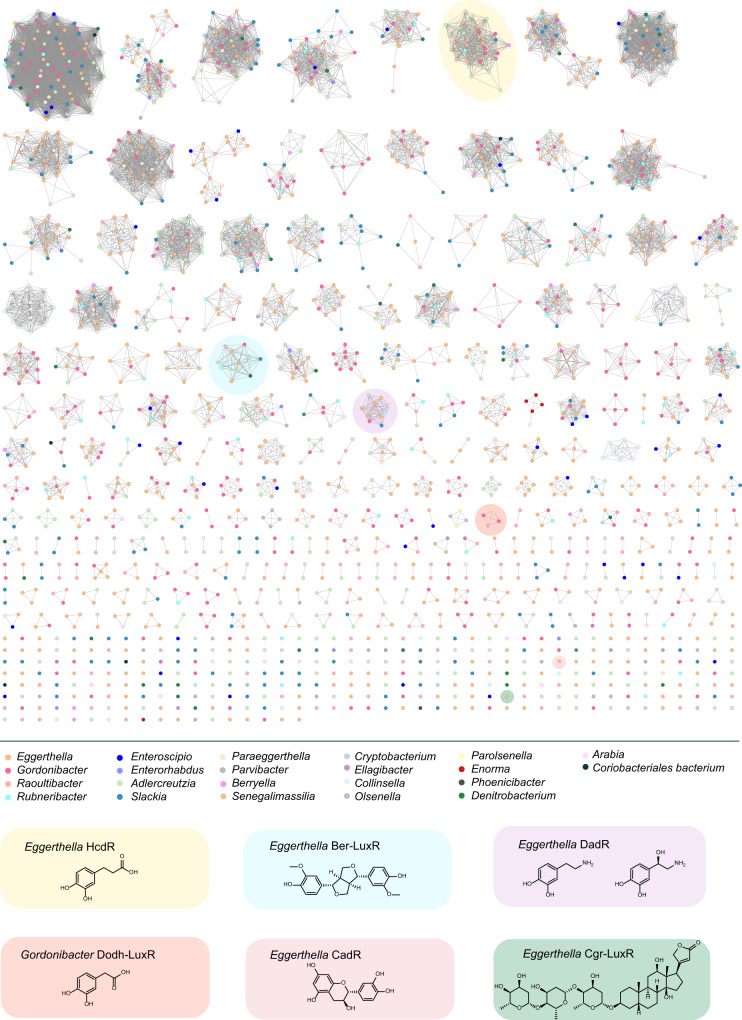
Sequence similarity network of Coriobacteriia 10–12-TM LuxR regulators reveals high diversity of these regulators. The SSN was constructed using Coriobacteriia protein sequences from IMG/M database containing a pfam00196 domain (Bacterial regulatory proteins, LuxR family) and predicted to have 10–12 N-terminal TM helices. Nodes represent proteins with 95% sequence identity. SSN displayed with an alignment score of 80. Nodes are coloured based on the bacterial genera that protein sequences belong to. Coloured clusters contain LuxR sequences that either are characterized in this study or are co-localized with characterized enzymes, with their representative LuxRs and potential sensing molecules displayed.

To delineate the distribution of TM helix LuxRs beyond *E. lenta*, we next queried other bacterial genomes. We found that LuxRs containing 10–12 N-terminal TM helices are widespread in Coriobacteriia, with multiple genera including *Eggerthella*, *Adlercreutzia*, *Gordonibacter*, and *Slackia* encoding >10 of these proteins per genome (Supplementary Data 3). Interestingly, bacteria containing >10 of such 10–12-TM LuxRs per genome are mostly Coriobacteriia (Supplementary Data 4). We further examined the relationship between the Coriobacteriia 10–12-TM helix LuxRs by building a sequence similarity network (SSN) using the web-based Enzyme Function Initiative-Enzyme Similarity Tool (EFI-EST)^34^ and Coriobacteriia protein sequences from IMG/M database which contain a pfam00196 domain (Bacterial regulatory proteins, LuxR family) and are predicted to have 10–12 N-terminal TM helices (Fig. 7). We then mapped our genetically characterized *E. lenta* DadR, HcdR and CadR, as well as other 10–12-TM LuxRs with postulated functions based on characterized co-localized enzymes, onto the SSN. We found some 10–12-TM LuxR groups, including DadR and HcdR, in multiple genera, while other 10–12-TM LuxRs are exclusive to a single genus, like CadR and the TM LuxR in the *cgr* operon, which are only found in *Eggerthella*. The vast majority of 10–12-TM LuxR clusters (>98%) cannot readily be assigned a function, suggesting this protein family has greatly diversified, potentially to adapt to different inducers and regulon sequences. Overall, the broad distribution and high diversity of 10–12-TM LuxRs in this taxon highlights an important biological role for these transcriptional regulators in this group of bacteria.

### Genomic engineering shows the *cgr* operon is necessary for Th17 cell activation by *E. lenta* in vivo

To further demonstrate the functional significance of developing a genetic toolkit for *E. lenta*, we sought to use our tools to study the effect of specific *E. lenta* genes on host biology. Specifically, we tested whether isogenic strains of *E. lenta* could recapitulate previously identified strain-variable traits. The strain-variable *cgr* (cardiac glycoside reductase) operon enables *E. lenta* to inactivate multiple cardenolides found in toxic plants, including the cardiac drug digoxin^8,9^. We recently discovered that the *cgr* operon also leads to the activation of intestinal Th17 cells, exacerbating mouse models of inflammatory bowel disease, potentially due to the metabolism of endogenous steroidal glycosides^16^. Colonization of mice with *E. lenta* strains that naturally vary in the presence of the *cgr* operon led to the expected differences in Th17 cell activation^16^; however, definitive results linking these genes to the observed phenotype are lacking due to the inability to construct targeted deletions and the marked differences in gene contents between strains^1^.

**Fig. 8:**
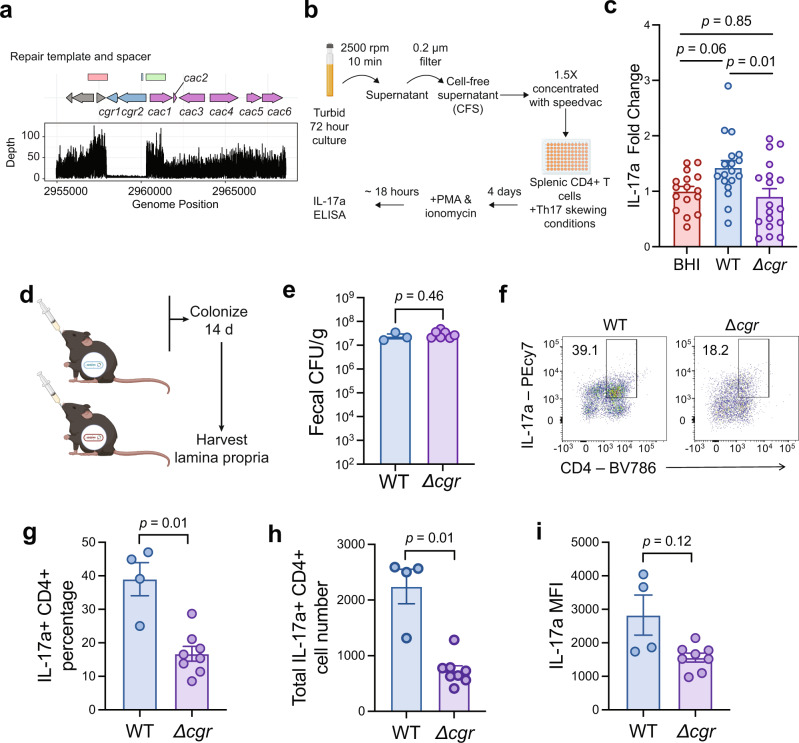
Genomic engineering and in vivo experiment show that the *cgr* operon is necessary for colonic Th17 induction by *E. lenta*. **a** Whole genome sequencing confirms the targeted deletion of the *cgr* operon. Additional reads flanking the deletion site are due to the presence of the editing plasmid in the long-read sequencing run, as indicated by red and green rectangles. **b** Schematic of in vitro Th17 skewing assay. **c** Fold-change of IL-17a levels relative to BHI control of CFS from *E. lenta* WT or *Δcgr*. **d** Experimental outline of mouse experiment. Germ-free C57BL/6J male mice ages 6–8 weeks were separated into groups and gavaged with *E. lenta* WT (*n* = 4) and *Δcgr* (n = 8). Bacteria were allowed to colonize for 2 weeks before the lamina propria was harvested. **e** No significant differences in *E. lenta* colonization on day 14. **f** Representative flow cytometry plots of colonic CD4^+^ IL-17a^+^ cells within the live CD3^+^ gate. Percentage of CD4 + IL-17a+ cells is displayed on the flow plot. **g** Percentage of colonic IL-17a^+^ CD4^+^ cells within the live CD3^+^ gate. **h** Total numbers of colonic IL-17a^+^ CD4^+^ cells within the live CD3^+^ gate. **i** Mean fluorescence intensity (MFI) of colonic IL-17a. All *p*-values are displayed and were calculated with one-way ANOVA tests with Tukey’s multiple correction or Welch’s t tests for two-way comparisons. Data represented as mean ± SD in **c** (BHI *n* = 16, WT and *Δcgr* *n* = 18 biological replicates), **e** (WT *n* = 3, *Δcgr* *n* = 7 because fecal pellets were unable to be collected from mouse 4 of the *E. lenta* WT group and mouse 8 of the *Δcgr* group) and **g**–**i** (WT *n* = 4, *Δcgr* *n* = 8). Each dot represents an individual biological replicate in **c** or an individual mouse in **e**, **g**, **h**, and **i**. Panels **e**–**i** show representative data from the first of two independent experiments. Source data are provided as a Source Data file.

Using our genomic engineering method, we deleted *cgr1* and *cgr2* in *E. lenta* DSM 2243 to generate a Δ*cgr* strain (Fig. 8a; Supplementary Fig. 6d). Long-read sequencing using the MinION platform confirmed the deletion of the *cgr* operon (Fig. 8a; Supplementary Fig. 8a). Additionally, we observed 120 single nucleotide polymorphisms, 25 small insertions (1–8 bp), and 81 small deletions (all 1 bp in length except one of 2 bp), likely due to a combination of errors in the reference genome and our de novo assembly (Supplementary Table 1). As the *cgr*-editing plasmid exhibited high segregation stability in the Δ*cgr* strain (Supplementary Fig. 1g), a non-targeting crRNA plasmid containing the same repair template as the editing plasmid was transformed into *E. lenta* DSM 2243 WT to generate an isogenic WT control for the Δ*cgr* strain.

Deletion of the *cgr* operon impaired the ability of *E. lenta* to induce Th17 cells in vitro and in gnotobiotic mice. Using our previously described in vitro assay for IL-17a induction^16^, we treated Th17-skewed splenic CD4^+^ T cells with cell free supernatant from the Δ*cgr* and WT strains (Fig. 8b). IL-17a levels were significantly lower in response to *E. lenta Δcgr* relative to WT controls (Fig. 8c). To assess the physiological relevance of these findings, we mono-colonized germ-free (GF) C57BL/6 J adult male mice with *E. lenta Δcgr* or WT for 2 weeks and harvested the colonic lamina propria to quantify Th17 cells (Fig. 8d). Both strains colonized the distal gut to similar levels: 3.0 × 10^7^ ± 1.0 × 10^7^ Δ*cgr* and 2.4 × 10^7^ ± 9.5 × 10^6^ WT CFUs/g stool (mean ± SD; Fig. 8e). As predicted, *E. lenta* WT led to a significant increase in colonic Th17 cells (Fig. 8f, g, h) and trending increase in IL-17a expression (Fig. 8i; Supplementary Fig. 8b) compared to the isogenic Δ*cgr* strain. These differences were not observed in the ileum (Supplementary Fig. 8c–e). Our findings provide more definitive evidence that the *cgr* operon is necessary for the induction of colonic Th17 cells and emphasize the utility of these new tools for the genetic manipulation of *E. lenta* to decipher the effects of bacterial genes on the host.

## Discussion

In this study, we develop a comprehensive set of genetic tools for a prominent, previously genetically intractable member of the human gut microbiota. Notably, we accomplish construction of reporter systems, inducible gene expression, and genome engineering in *E. lenta*. To our knowledge, this represents the first successful attempt to genetically manipulate members of the Coriobacteriia taxon systematically. Our study also highlights remaining challenges in developing genetic tools for *E. lenta*. The large variations in the transformation efficiency observed for different *E. lenta* strains is a current limitation, and more work is needed to understand this phenomenon and extend these technologies to other isolates.

We used these tools to decipher the regulation of *E. lenta* catechol dehydroxylases, providing initial insights into exquisitely specific induction of these enzymes. In this work, we characterized three endogenous promoters (P_*dadh*_, P_*cadh*_, and P_*hcdh*_) that are induced by dopamine/norepinephrine, (+)-catechin, and hydrocaffeic acid, respectively. The induction specificity of each promoter for different catechols matches the substrate specificity of the corresponding catechol dehydroxylase enzyme. The congruence of enzyme substrates and promoter inducers may apply to the many other *E. lenta* enzymes regulated by TM LuxRs; if so, one could envision uncovering substrates of uncharacterized enzymes by combining high-throughput screening of small molecules with construction of TM LuxR-based reporter systems.

Perhaps most importantly, we revealed that a unique class of LuxR-type transcriptional regulators is the key activator underlying the induction of catechol dehydroxylase enzymes in response to their substrates. These 10–12-TM LuxRs, like canonical LuxRs^28^, possess a C-terminal HTH DBD. Whereas canonical LuxRs commonly possess a cytoplasmic N-terminal domain that binds to a signalling molecule^28^, these TM LuxRs have a unique N-terminal 10–12-TM domain. While the molecular mechanisms underlying their functions are unclear, we speculate that  the N-terminal TM domain or its extracellular loops may interact with substrates and somehow relay the signal to its cytoplasmic DBD, resulting in expression of its associated regulon (Fig. 6d). The striking diversification of the regulator family within Coriobacteriia along with the tight expression control and high inducibility of their regulated promoters suggests this group of bacteria has evolved transcriptional machinery specialized for recognition of individual substrates. Further biochemical, bioinformatic and structural studies of 10–12-TM LuxRs are needed to elucidate the detailed mechanisms underlying the function of these regulators, the signalling molecules they recognize, and their evolutionary origins. We also anticipate using similar genetic approaches to characterize DadS, another putative transcriptional regulator encoded next to *dadR*. Though we currently cannot rule out a role for this protein in regulation of *dadh*, its genomic context suggests DadS may be involved in regulating a distinct set of downstream genes, including a TorD family molecular chaperone and various ferredoxins that could be important for dopamine metabolism^35^, in response to dopamine (Supplementary Fig. 3b). Moreover, the lack of corresponding LysR homologs in any other LuxR-containing *E. lenta* gene clusters (Supplementary Fig. 7) indicates these proteins are likely not involved in regulating most metabolic activities of *E. lenta*.

Catechol is a structural motif found in numerous compounds in the human gut, including dietary phytochemicals and neurotransmitters. Identifying three members of this emerging family of 10–12-TM LuxRs and their roles in regulating catechol metabolism highlights the interplay between bacterial regulatory networks, metabolic activities, and catechols in the human gut. Among the catechols present in this environment, the neurotransmitters dopamine and norepinephrine are important host- and gut microbiota-derived molecules that contribute to bidirectional host-microbial signalling^36^. It remains unaddressed why *E. lenta* has evolved specific systems to respond to and metabolize host neurotransmitters, and whether neurotransmitter metabolism regulates *E. lenta* behaviours such as colonization in the gut, and/or impacts host health. Future studies could combine genetic manipulation with animal colonization experiments to assess the physiological relevance of the neurotransmitter metabolism in *E. lenta*, and potentially target and rewire the *E. lenta* dopamine metabolism pathway to impact host health.

The ability to manipulate genes in individual members of the gut microbiota is essential to provide insights into their effects on the host. By performing targeted deletion of the *cgr* genes in *E. lenta* DSM 2243 and examining its effect on germ-free mice, we specifically connect this operon to activation of host Th17 cells. Our success in applying this genetic toolkit to in vivo experiments sets the stage for future dissection of metabolic functions and host-microbe interactions involving this prevalent human gut microbe. In particular, the genome engineering and inducible expression platforms we have developed allow for targeted manipulation of individual *E. lenta* gene activities. In the future, we envision these tools will be applied to other gut Coriobacteriia to identify bacterial functions that contribute to host pathophysiology. Ultimately, understanding how *E. lenta* and other gut Coriobacteriia modulate host immunity and diseases may reveal future opportunities for bacterial engineering to improve host health.

## Methods

### Ethical statement

This research complies with all relevant ethical regulations. Mouse experiments were approved by the University of California, San Francisco Institutional Animal Care and Use Committee.

### Bacterial culture

Routine culturing of *E. lenta* and *Gordonibacter* was done under anaerobic conditions (Coy Lab Products) with an atmosphere of 2 to 4% H_2_, 2 to 4% CO_2_, and N_2_ as the balance. *E. lenta* cultures were grown in BHI+ medium (BHI with 1% w/v arginine) or BHIrf medium (BHI with 1% w/v arginine and 10 mM sodium formate) at 37 °C. For *E. lenta* DSM 2243 transformed with kanamycin-resistance plasmids, cultures were grown in BHI+ or BHIrf supplemented with 100 μg/mL of kanamycin. For *E. lenta* DSM 2243 transformed with a tetracycline-resistance plasmid, cultures were grown in BHI+ or BHIrf supplemented with 20 μg/mL of tetracycline.

### Plasmids and DNA oligonucleotides

Cloning work was performed using Gibson Assembly (NEB, catalogue number E2611S) or NEBuilder HiFi DNA Assembly (NEB, catalogue number E2621S). The oligonucleotide sequences used for this work are listed in Supplementary Data 5, and the sequences for the genetic parts involved in this study are listed in Supplementary Data 6.

To construct the entry *Eggerthella-E. coli* shuttle plasmids pXD69m1 and pXD69m2, genomic DNA (gDNA) of *E. lenta* DSM 11863, A2, and DSM 11767 was prepared from an anaerobic 48 h liquid BHI+ culture using the DNeasy UltraClean Microbial kit (QIAGEN catalogue number 12224-50). The backbone of the shuttle plasmids was amplified from *E. lenta* DSM 11863 gDNA. The *E. lenta* tetracycline resistance gene *tetW* and its promoter were amplified from A2 gDNA. The *E. lenta* kanamycin resistance gene *aphA* and its promoter were amplified from DSM 11767 gDNA. The backbone and *E. lenta* antibiotic gene were ligated with *E. coli bla* and replicon amplified from plasmid pCT5-bac2.0^23^. pCT5-bac2.0 was a gift from Claudia Schmidt-Dannert (Addgene plasmid #119872; http://n2t.net/addgene:119872; RRID:Addgene_119872). To construct plasmid pXD68Kan2, the RSF1010-containing backbone was amplified from plasmid pAM5409^20^, and was ligated with *E. lenta aphA* gene. pAM5409 was a gift from Susan Golden (Addgene plasmid #132662; http://n2t.net/addgene:132662; RRID:Addgene_132662).

To further engineer the shuttle plasmids, a gene fragment containing two sets of tandem terminators was synthesized (Twist Bioscience) and inserted into EcoRI-digested pXD69m2 to make pXD70entry. In all of the pXD69m2-derived plasmids, functional cassettes were cloned in between the two sets of terminators in pXD70entry to prevent possible interference between the expression cassettes and other genes on the plasmid. To construct *lacZ* reporter plasmids, *lacZ* gene was amplified from *E. coli* MG1655 gDNA, and cloned into pXD70entry for promoterless pXD70LacZ1, or with respective promoters P_*csd*_ and P_*degV*_ to make pXD70LacZ3 and pXD70LacZ4, respectively. *E. lenta* endogenous regulatory elements P_*dadh*_, *dadR/S-*P_*dadh*_, *cadR-*P_*cadh*_, P_*cadh*_ and P_*hcdh*_ were amplified from *E. lenta* A2 gDNA and cloned upstream of the *lacZ* gene to make pXD70LacZ2, pXD70LacZ7, pXD70LacZ8, pXD70LacZ8.1 and pXD70LacZ9, respectively. P_*hcdR*_-*hcdR* was amplified from *E. lenta* A2 gDNA and ligated to pXD70LacZ9 to construct pXD70LacZ9.1.

For the cumate-inducible *lacZ* reporter plasmids, the regulatory region containing the *cymR* gene and promoters was amplified from pCT5-bac2.0^23^ and cloned upstream of *lacZ* gene to make pXD70CT5. Further, a gene fragment that harbours P_*degV*_-controlled *cymR*, P_*csd*_ inserted with a cumate operator sequence, and a terminator in between, was synthesized and cloned upstream of *lacZ* gene to make pXD70CT3. To construct pXD70CT5.1, another cumate operator sequence was further introduced into pXD70CT5. PCR amplification using primer pairs oXD294/oXD440 and oXD361/oXD442 were performed with pXD70CT5 plasmid and cloned into pXD70entry by Gibson Assembly to make pXD70CT5.1.

To construct the Dadh-expressing plasmid pXD70DA7, the coding sequences of DadR/DadS, DadhABC and the flanked promoter region were amplified from *E. lenta* A2 and cloned into the pXD70entry backbone. In addition, the coding sequence of A2 DadhABC was cloned to replace the *lacZ* gene in pXD70CT5 to construct cumate-inducible Dadh plasmid pXD70DA9. Primer pairs oXD401/oXD989 and oXD990/oXD351 were used to amplify Dadh(A2, R506S) from pXD70DA7, and the DNA fragments were cloned into the pXD70entry backbone to construct pXD70DAmt1.

For the crRNA cloning, a primer set (oXD384/oXD292) was first used to amplify the pXD70CT5 backbone without the ribosome binding site and *lacZ* gene. The primers containing a repeat-pseudospacer-repeat organization were annealed and cloned into the pXD70CT5 backbone using Gibson Assembly to make pseudo-crRNA plasmid pXD71Cas5. A *dadR*-targeting crRNA plasmid pXD71Cas3 was constructed similarly to contain a repeat-*dadR* spacer-repeat. To reduce the residual leakiness of crRNA expression, another cumate operator sequence was further introduced into both plasmids. PCR amplification using primer pairs oXD291/oXD440 and oXD361/oXD442 were performed with both pXD71Cas5 and pXD71Cas3 plasmids, and ligated by Gibson Assembly to make pXD71Cas10.0 and pXD71Cas10.1, respectively, which contain two tandem cumate operator sequences. For genome editing, the repair template was inserted in place of the *E. coli* ampicillin-resistance gene *bla* into pXD71Cas10.1, as we observed that the *E. lenta aphA* gene is also sufficient for selecting transformants of *E. coli*. The pXD71Cas10.1 backbone was amplified with primers oXD529 and oXD530 to remove the *bla* gene. Homologous arms 1-kb upstream and 1-kb downstream of *dadR* coding sequences were amplified from *E. lenta* DSM 2243 gDNA and ligated into the pXD71Cas10.1 backbone to make plasmid pXD71Cas10.1RT.

The cloning of other crRNA plasmids was performed by Golden Gate Assembly using type IIS restriction enzyme PaqCI (NEB, catalogue number R0745) and T4 DNA Ligase (NEB, catalogue number M0202). The vector pXD71Cas10.0 possesses two PaqCI restriction sites in the pseudospacer region and could be digested by PaqCI to enable the cloning of annealed oligonucleotides. The primers carrying the spacers of interest and 4-bp overhangs, were annealed and phosphorylated in a single 50 µL reaction with 5 µL 10x T4 Ligation Buffer (NEB, catalogue number B0202S) and 1 µL T4 Polynucleotide kinase (NEB, catalogue number M0201S) by incubating at 37 °C for 2 h, 95 °C for 5 min, and ramping down to 20 °C at 2 °C/min. The mixtures were diluted 1:20 in water and 1 µL was used to ligate to 75 ng of pXD71Cas10.0 vector, adding 2 µL 10x T4 Ligation Buffer, 0.5 µL PaqCI (R0745; 10 unit/μL), 0.25 µL PaqCI Activator (20 μM), 0.5 μL T4 DNA Ligase (M0202, 400 unit/μL) and water to a total volume of 20 µL. The ligation was carried out at 37 °C for 1 h and was stopped by incubating at 65 °C for 5 min. 2 µL of the ligation were used to transform into TOP10 competent *E. coli*.

The DadR-encoding plasmids were derived from tetracycline-resistance vector pXD69m1. To introduce tandem terminators into pXD69m1, a *lacZ-*expressing tetracycline-resistance plasmid pXD70Tet(LacZ3) was first constructed. The P_*csd*_-*lacZ* cassette and the flanking tandem terminators were amplified from pXD70LacZ3 using primers oXD271 and oXD348, and ligated to pXD69m1 backbone amplified using primers oXD536 and oXD329 to make pXD70Tet(LacZ3). The backbone of pXD70Tet(LacZ3) was amplified using primers oXD291 and oXD292, and subsequently ligated with P_*dadR*_-DadR, or DadR mutants amplified from *E. lenta* A2 gDNA, P_*dadh*_-Dadh(A2) and DadR-P_*dadh*_-Dadh(A2) amplified from pXD70DA7, and P_*hcdR*_-HcdR, P_*cadR*_-CadR amplified from *E. lenta* DSM 2243 gDNA to construct pXD70Tet(DadR), pXD70Tet(DadR(ΔDBD)), pXD70Tet(DadR(ΔTM)), pXD70Tet(DadR(linker)), pXD70Tet(FLAG-DadR), pXD70Tet(FLAG-DadR(ΔDBD)), pXD70Tet(FLAG-DadR(ΔTM)), pXD70Tet(FLAG-DadR(linker)), pXD70Tet(DadH), pXD70Tet(DadHR), pXD70Tet(HcdR) and pXD70Tet(CadR), respectively.

### Bacterial transformations

Transformation of various *E. lenta* strains and *Gordonibacter* species was carried out using electroporation. For preparation of *E. lenta* or *Gordonibacter* electrocompetent cells, 1 mL of 48 h saturated cultures was inoculated into 100 mL of BHI+ medium and grown at 37 °C to OD_600_ 0.2–0.4. Cultures were chilled on ice for 20 min, centrifuged, washed three times with 10 mL of ice-cold water, and washed once with 5 mL of 10% ice-cold aqueous glycerol solution (or other electroporation buffer as indicated elsewhere). The cell pellets were suspended in 2–4 mL of 10% aqueous glycerol solution (or other electroporation buffer as indicated elsewhere).

For bacterial transformation, 100 µL aliquots of *E. lenta* or *Gordonibacter* electrocompetent cells were electroporated using a MicroPulser Electroporator (BioRad) with 100–2000 ng of plasmid at 2.5 kV voltage, using 0.1-mm gap width electroporation cuvettes (VWR). 1 mL of BHIrf medium was immediately added to the electroporated cells and transferred to 1.7 mL Eppendorf tubes. These tubes were brought to anaerobic chamber (Coy) and incubated at 37 °C anaerobically for 3 h. Part or all of the transformations were plated onto BHI+ or BHIrf agar with the appropriate antibiotic (100 μg/mL kanamycin or 20 μg/mL tetracycline, or both) and grown anaerobically for 3–4 days at 37 °C. For transformation of *E. lenta* AB8n2, BHIrf agar with 150 μg/mL kanamycin was used.

### Plasmid maintenance assay

*E. lenta* DSM 2243 cultures harbouring different plasmids were grown to saturation in BHIrf media supplemented with either 100 μg/mL of kanamycin or 20 μg/mL of tetracycline. For *E. lenta* cultures harbouring pXD69m1, pXD69m2 or pXD68Kan2, the saturated cultures were first inoculated 1:500 into BHIrf medium and the resulting diluted cultures were further diluted 1:500 into BHIrf medium. For the *E. lenta* Δ*cgr* strain, the saturated cultures were first inoculated 1:500 into BHIrf medium, the resulting diluted cultures were further diluted 1:500 into BHIrf medium, and then a final 1:1000 dilution into BHIrf medium was performed. The diluted cultures were grown in the absence of antibiotics anaerobically for 2 days. The saturated cultures were then serially 10-fold diluted onto BHIrf agar plates with or without selective antibiotics and grown for 2–3 days, and CFU counts were performed. Each culture was passaged five times using the procedure described above to quantify plasmid maintenance. Results are presented as the ratio of CFUs from the BHIrf agar with antibiotic (selective) over CFUs from the BHIrf agar without antibiotic (permissive).

### *lacZ* reporter assay

The β-galactosidase assay was adapted from a previously described protocol^37^. For measurement of β-galactosidase activity in *E. lenta* DSM 2243 WT and pXD70LacZ1–LacZ4, saturated cultures were inoculated 1:100 in fresh BHI+ medium (with kanamycin for pXD70LacZ1–LacZ4 constructs) in Hungate tubes and grown to an OD_600_ of 0.2–0.5. 1 mL of each culture was harvested by centrifugation and resuspended in 1 mL of lysis buffer [2 mg/mL lysozyme dissolved in a mixture of 50 µL BugBuster 10X (Novagen, catalogue number 70921-5) and 950 µL Z-buffer (60 mM Na_2_HPO_4_ • 7H_2_O, 40 mM NaH_2_PO_4_ • H_2_O, 10 mM KCl, 1 mM MgSO_4_, 50 mM β-mercaptoethanol), pH 7.0]. The cell suspensions were lysed by incubating at 37 °C for 30 min. After the incubation, 200 µL of 4 mg/mL *o*-nitrophenyl-β-D-galactoside (ONPG) solution in Z-buffer was added and the reaction mixture was incubated at 30 °C with constant agitation. The reaction was stopped by applying 500 μL of 1 M Na_2_CO_3_. The lysozyme precipitates at basic pH, so after centrifugation (15000 × *g*, 5 min, 4 °C) to remove cell debris, the absorbance at 420 nm and at 550 nm was immediately measured using a Beckman Coulter DU730 UV–Vis Spectrophotometer. The time until absorbance measurement was recorded. A blank containing all of the components mentioned above, excluding cells, was used. The β-galactosidase activity of these *E. lenta* cultures was normalized to the cell density and was presented as Miller Units.

The β-galactosidase activity assay for all other *lacZ* reporters was performed in 96-well plates (Corning) using Beta-Glo® Assay System (Promega, E4740). For cumate-inducible *lacZ* constructs, saturated cultures harbouring either pXD70CT3 or pXD70CT5 were inoculated 1:20 in fresh BHIrf medium with 100 μg/mL kanamycin in 96-well plates. Cumate was first dissolved in dimethylformamide (DMF) to make a 500 mM stock solution, and then 50 μL of the 500 mM stock solutions was further diluted 1:40 into 1950 μL of BHIrf to make a 12.5 mM stock solution. Further dilution of the 12.5 mM stock solution in BHIrf yielded 25x stock solutions for lower concentrations. 8 μL of 25× cumate stock solutions of different concentrations or 8 μL of BHIrf were added to 192 μL of the diluted cultures in triplicate, and the cultures were incubated anaerobically at 37 °C for 20–24 h. BHIrf medium was used as blank. The OD_600_ of the overnight cultures was measured using a Synergy HTX Multi-Mode Microplate Reader (BioTek). Then 20 μL of culture was incubated with 20 μL of Beta-Glo reagent with constant agitation at 30 °C for 45 min. After the incubation, the luminescence intensity of the reaction mixture was measured using Microplate Reader (BioTek) (gain, 135; integration time, 1 s). Blank OD_600_ and luminescence intensity were subtracted from the sample readout. To quantify the specific β-galactosidase activity of each sample, luminescence intensity was normalized to the culture endpoint OD_600_.

For IPTG-inducible pXD70LacZ6 reporter strain, saturated cultures were inoculated 1:20 in fresh BHIrf medium with 100 μg/mL kanamycin in 96-well plates. A 500 mM IPTG stock solution was diluted 1:20 in BHIrf to make a 25 mM stock solution, and further dilution of the 25 mM stock solutions in BHIrf yielded 25× stock solutions for lower IPTG concentrations. 8 μL of 25x IPTG stock solutions, or 8 μL of BHIrf were added to 192 μL of the diluted cultures in triplicate, and the cultures were incubated anaerobically at 37 °C for 20–24 h. The following treatments were the same as described for cumate-inducible *lacZ* reporters.

For catechol-inducible *lacZ* reporter strains, saturated cultures were inoculated 1:20 in fresh BHIrf medium with 100 μg/mL kanamycin in 96-well plates. Dopamine and norepinephrine were dissolved in BHIrf to make a 100 mM stock solution. Hydrocaffeic acid was dissolved in H_2_O to make a 100 mM stock solution. (+)-catechin was first dissolved in DMF to make a 500 mM stock solution and then further diluted 1:10 in H_2_O to make a 50 mM (+)-catechin stock solution. Further dilution of the 100 mM or 50 mM catechol stock solutions in BHIrf yielded 50x stock (pXD70LacZ2, pXD70LacZ7, pXD70LacZ8 and pXD70LacZ8.1 assays) or 12.5× stock solutions (pXD70LacZ9 and pXD70LacZ9.1 assays) for lower catechol concentrations. 4 μL of 50× or 16 μL of 12.5x catechol stock solutions, or the same volume of vehicle were added to 196 μL or 184 μL of the diluted cultures in triplicate, and the cultures were incubated anaerobically at 37 °C for 20–24 h. The following treatments were the same as described for cumate-inducible *lacZ* reporters.

### Expression and activity of *E. lenta* A2 dopamine dehydroxylases in *E. lenta* DSM 2243

*E. lenta* DSM 2243 cultures harbouring pXD70DA7, pXD70DAmt1 or pXD70DA9 were grown to saturation in BHIrf media with kanamycin. The saturated cultures were then inoculated 1:50 into 200 μL of BHIrf with 100 μg/mL kanamycin in quadruplicate in 96-well plates. 1 mM dopamine was then added to the pXD70DA7 and pXD70DAmt1 cultures. 1 mM dopamine with or without 50 μM cumate was added to the pXD70DA9 cultures. Plates were incubated at 37 °C anaerobically for 48 h. Next, the endpoint OD_600_ of each culture was measured using a Synergy HTX Multi-Mode Microplate Reader (BioTek). The plates were then centrifuged (3220 × *g*, 10 min, 4 °C), and the supernatants were harvested. For each sample, 20 µL of the supernatant was diluted 1:10 with 180 µL of LC-MS grade (Honeywell) methanol, and 40 µL of the resulting mixture was then diluted 1:5 with 160 µL of Milli-Q water. The amount of dopamine and *m*-tyramine production in the diluted mixtures were quantified measured using ultra-performance liquid chromatography tandem mass spectrometry (UPLC-MS/MS).

UPLC-MS/MS was conducted using a Waters Acquity UPLC H-Class System (Waters Corporation), and Waters Xevo TQ-S (Waters Corporation) instrument. 2 µL of each sample was injected onto a CORTECS T3 Column (120 Å, 2.7 µm, 2.1 mm × 100 mm, Waters Corporation). The flow rate was 0.5 mL/min using solvent A = 0.1% formic acid in H_2_O and solvent B = 0.1% formic acid in acetonitrile (Honeywell). The column temperature was maintained at 40 °C. The following gradient was applied: 0–1 min at 100% A isocratic, 1.0–2.0 min at 0–90% B, 2.0–2.5 min at 90% B isocratic, 2.5–2.75 min at 90–0% B, 2.75–3.50 min at 0% B isocratic. MS detection was performed using electron spray ionization in positive mode (ESI+) (capillary voltage, 3.20 kV; cone voltage, 29 V; source offset voltage, 50 V; desolvation temperature, 200 °C; desolvation gas flow, 800 L/h; cone gas flow, 150 L/h; nebulizer, 7.0 bar). The masses of dopamine (precursor ion *m*/*z* = 154.1119, daughter ion *m*/*z* = 137.0550; cone voltage 30 V; collision energy 8 V), and *m-*tyramine (precursor ion *m*/*z* = 138.1119, daughter ion *m*/*z* = 121.1114; cone voltage 8 V; collision energy 10 V) were monitored. Concentrations of dopamine and *m-*tyramine were quantified using calibration curves.

### Measuring the impacts of DadR on dopamine metabolism of *E. lenta* DSM 2243 expressing Dadh(A2)

Plasmid pXD70Tet(DadH) encoding the active Dadh variant Dadh(A2) under native promoter P_*dadh*_, and plasmid pXD70Tet(DadHR) encoding Dadh(A2) under native promoter P_*dadh*_ and DadR were transformed into the Δ*dadR* mutant and DSM 2243 WT by electroporation. The *E. lenta* strains were inoculated and grown to saturation in BHIrf media with 20 μg/mL tetracycline. The saturated cultures were then inoculated 1:20 into 200 μL of BHIrf with 20 μg/mL tetracycline in quadruplicate in 96-well plates. 1 mM dopamine was then added to each culture. Plates were incubated at 37 °C anaerobically for 72 h. Next, the endpoint OD_600_ of each culture was measured using a Synergy HTX Multi-Mode Microplate Reader (BioTek). The plates were then centrifuged (3220 × *g*, 10 min, 4 °C), and the supernatants were harvested. For each sample, 20 µL of the supernatant was diluted 1:10 with 180 µL of LC-MS grade (Honeywell) methanol, and 40 µL of the resulting mixture was then diluted 1:5 with 160 µL of Milli-Q water. The amount of dopamine and *m*-tyramine production in the diluted mixtures were quantified measured using UPLC-MS/MS as described above.

### Assays for measuring (+)-catechin and hydrocaffeic acid metabolism of *E. lenta* DSM 2243 WT and engineered strains

*E. lenta* DSM 2243 WT strain and mutant strains were grown to saturation in BHIrf media, and the complementation strains were grown to saturation in BHIrf with 20 μg/mL tetracycline. The saturated WT and mutant cultures were then inoculated 1:50 into 200 μL of BHIrf in triplicate or quadruplicate in 96-well plates. The saturated complementation strain cultures were inoculated 1:20 into 200 μL of BHIrf supplemented with 20 μg/mL tetracycline in triplicate or quadruplicate in 96-well plates. 2 mM hydrocaffeic acid or 1 mM (+)-catechin was then added to the cultures. Plates were incubated at 37 °C anaerobically for 48 h. Next, the endpoint OD_600_ of each culture was measured using a Synergy HTX Multi-Mode Microplate Reader (BioTek). The plates were then centrifuged (3220 × *g*, 10 min, 4 °C), and the supernatants were harvested. To detect hydrocaffeic acid and dehydroxylation product 3-(3-hydroxyphenyl)propanoic acid (*m*-HPPA), 20 µL of the supernatant was diluted 1:10 with 180 µL of LC-MS grade (Honeywell) methanol, and 20 µL of the resulting mixture was then diluted 1:10 with 180 µL of Milli-Q water. To detect (+)-catechin metabolism derivatives, 20 µL of the supernatant was diluted 1:10 with 180 µL of LC-MS grade (Honeywell) methanol, and 100 µL of the resulting mixture was then diluted 1:1 with 100 µL of Milli-Q water. The amount of catechols and products in the diluted mixtures were quantified using UPLC-MS/MS.

UPLC-MS/MS was conducted using a Waters Acquity UPLC H-Class System (Waters Corporation), and Waters Xevo TQ-S (Waters Corporation) instrument. 1 µL of each sample was injected onto a CORTECS T3 Column (120 Å, 2.7 µm, 2.1 mm × 100 mm, Waters Corporation). The same chromatographic conditions used for dopamine metabolite detection were used for both hydrocaffeic acid and (+)-catechin metabolite detection.

MS detection of hydrocaffeic acid and *m*-HPPA was performed using electron spray ionization in negative mode (ESI−) (capillary voltage, 3.20 kV; cone voltage, 29 V; source offset voltage, 50 V; desolvation temperature, 200 °C; desolvation gas flow, 800 L/h; cone gas flow, 150 L/h; nebulizer, 7.0 bar). The masses of hydrocaffeic acid (precursor ion m/z = 181.0898, daughter ion m/z = 137.1128; cone voltage 2 V; collision energy 12 V), and *m-*HPPA (precursor ion m/z = 165.0919, daughter ion m/z = 106.0780; cone voltage 36 V; collision energy 20 V) were monitored. Concentrations of hydrocaffeic acid and *m-*HPPA were quantified using calibration curves.

MS detection of (+)-catechin and its metabolites was performed using electron spray ionization in negative mode (ESI–) (capillary voltage, 3.20 kV; cone voltage, 29 V; source offset voltage, 50 V; desolvation temperature, 200 °C; desolvation gas flow, 800 L/h; cone gas flow, 150 L/h; nebulizer, 7.0 bar). The masses of (+)-catechin (precursor ion m/z = 289.2, daughter ion m/z = 109.1; cone voltage 30 V; collision energy 20 V), benzyl ether reduced catechin (precursor ion m/z = 291.2, daughter ion m/z = 123.1; cone voltage 30 V; collision energy 20 V), benzyl ether reduced, dehydroxylated catechin (precursor ion m/z = 275.2, daughter ion m/z = 107.1; cone voltage 30 V; collision energy 20 V) were monitored.

### Genomic engineering

Genomic self-targeting of *E. lenta* DSM 2243 was achieved by electroporating competent cells with 200 ng of pXD71Cas10.1 expressing the *dadR*-targeting spacer, or pXD71Cas10.0 which contains a nontargeting pseudospacer as control. Cells were plated onto BHI+ agar plates containing 100 μg/mL kanamycin and grown for 3–4 days. Single colonies were then grown in liquid BHI+ medium containing 100 μg/mL kanamycin to saturation. Each saturated culture was then serially 10-fold diluted onto BHI+ agar plates containing 100 μg/mL kanamycin with or without 50 μM inducer cumate and grown for 3–4 days. The surviving colonies of pXD71Cas10.1 on cumate-containing plates were then analyzed individually using colony PCR.

For genome editing to generate a targeted deletion in *E. lenta* DSM 2243, a homology-directed repair (HDR) template was cloned into the self-targeting crRNA plasmid. A DNA repair template with about 1000 bp of the upstream and downstream regions flanking the deletion region was designed to introduce the desired deletion. 500–2000 ng of plasmid was used for electroporation, and the transformants were selected on BHI+ or BHIrf agar with 100 μg/mL kanamycin. The transformants obtained were PCR-screened to confirm the presence of desired mutations. Single clones that contain desired mutations were inoculated into BHI+ or BHIrf medium with 100 μg/mL kanamycin and 50 μM cumate, and the 2-day cultures were diluted and spread onto BHI+ or BHIrf agar plates containing 100 μg/mL kanamycin and 50 μM inducer cumate. The clones selected by crRNA-induced condition were then PCR-screened and sequenced to confirm the absence of WT sequence and the presence of designed mutations.

### Culturing *E. lenta* with catechols, RNA extraction, and RT-qPCR experiments

Saturated cultures of *E. lenta* DSM 2243 WT, mutant strains, and complementation strains were inoculated into 30 mL of BHIrf medium and grown to OD_600_ of 0.3–0.6. Antibiotic (20 μg/mL tetracycline) was added for the complementation strains. Then each strain was divided into 6 tubes of 5 mL cultures. For *E. lenta* DSM 2243 WT, Δ*dadR* mutant, and Δ*dadR* complementation strains, cultures were exposed to either 50 μL of dopamine (100 mM in BHIrf) or 50 μL of vehicle (BHIrf) in triplicate. For *E. lenta* DSM 2243 WT and Δ*hcdR* mutant, cultures were exposed to either 100 μL of hydrocaffeic acid (100 mM in H_2_O) or 100 μL of vehicle (H_2_O). For *E. lenta* DSM 2243 WT and Δ*cadR* mutant, cultures were exposed to either 100 μL of (+)-catechin (50 mM in 10% [v/v] DMF/BHIrf) or 100 μL of vehicle (10% [v/v] DMF/BHIrf). Then cultures were incubated anaerobically at 37 °C for 2–3 h. Cell pellets were then harvested by centrifugation (3220 g, 10 min, 4 °C), resuspended in 800 µL of TRIzol reagent (Invitrogen, catalogue number 15596-026), and used for RNA extraction immediately or stored in a freezer at −80 °C until RNA extraction.

Total RNA was isolated first by bead beating the TRIzol cell suspensions for 2.5 min twice to lyse cells using Mini-Beadbeater-16 (BioSpec) and ZR BashingBead Lysis Tubes (0.1 and 0.5 mm) (Zymo Research, catalogue number S6012-50), and then using the Zymo Research Direct-Zol RNA MiniPrep Plus kit (catalogue number R2070) according to the manufacturer’s protocol. Briefly, an equal volume of ethanol was mixed with lysates and the mixture was transferred to the provided spin column and centrifuged to remove flow-through. The column was transferred to a new collection tube. To the column, 80 µL of DNase mix (5 µL of DNase I (6 U/µL) and 75 µL of DNA Digestion Buffer) was added, and the column was incubated at room temperature for 15 min. Next, 400 µL of RNA PreWash was applied to the column twice, followed by 700 µL of RNA Wash Buffer. The column was carefully transferred to an RNase-free tube. Then, 50 µL of RNase-free water was added, and the column was incubated at room temperature for 1 min and centrifuged to collect total RNA. Then a second solution-phase DNase treatment was performed using Promega RQ1 RNase-Free DNase (catalogue number M6101), adding 2 µL of RQ1 RNase-Free DNase 10X Reaction Buffer, 1 µL of DNase, and 15 µL of RNase-free water to a 2 µL aliquot of total RNA. This mixture was incubated at 37 °C for 30 min, after which 2 µL of RQ1 DNase Stop Solution was added. The resulting mixture was incubated at 65 °C for 10 min to terminate the reaction and inactive the DNase. All, or a portion of, the treated RNA was used for the subsequent RT-qPCR.

From total RNA, complementary DNA synthesis and PCR amplification were performed using the Luna Universal One-Step RT-qPCR Kit according to the manufacturer’s protocol (NEB, catalogue number E3005S). Assays were performed using a Thermocycler C1000 CFX96 Real-Time System (Bio-Rad). The primers used for amplification are listed in the Supplementary Data 5. Fold changes in *dadh*, *hcdh* and *cadh* transcript levels were calculated using the ΔΔC_T_ method.

### Western blotting

Saturated cultures of *E. lenta ΔdadR* strains harbouring various N-terminal FLAG-tagged DadR or DadR mutant constructs were inoculated 1:20 in fresh BHIrf medium with 20 μg/mL tetracycline and were either incubated with vehicle (BHIrf) or 1 mM dopamine at 37 °C for 40 h. Cell pellets were then harvested by centrifugation (3220 × *g*, 10 min, 4 °C), and heated in Laemmli Sample Buffer with 2-mercaptoethanol at 98 °C for 10 min. Standard Western blotting analysis was performed using 10–20% SDS-PAGE gels with primary Anti-FLAG antibody (1:1000, Sigma, F1804-50UG) incubation at 4 °C overnight. Secondary antibody incubations were done at room temperature for 1 h using anti-Mouse IgG–Peroxidase antibody (1:4000, Sigma, A4416-.5 ML) and then imaged using an Azure Imaging Systems (Azure Biosystems).

### Whole Genome Sequencing

High molecular weight DNA was extracted from 2 mL 72 h cultures of *Δcgr E. lenta* grown in BHI+ using the MagAttract HMW DNA Kit (Qiagen) following the manufacturer’s instructions with the following modifications. Bacterial pellets were resuspended in 450 µL of Buffer P1, transferred to a Lysing Matrix E 2 mL tube (MP), and lysed using a BioSpec Mini-Beadbeater-96 for 30 s. 200 µL of the supernatant was transferred to a clean tube. 40 µL of 100 mg/mL lysozyme was added, and the mixture was incubated at 37 °C with shaking at 900 rpm for 2 h. After all other steps were carried out per the protocol, HMW DNA was eluted in 100 µL of nuclease-free water.

Genomic library preparation was performed using the ONT Native Barcoding Kit (SQK-NBD112.24). Sequencing was performed using the MinION sequencer with R10.4 flow cell. Data processing and demultiplexing were performed by MinKNOW core v5.0.0, using Guppy v6.0.7 for base calling in “fast” mode.

A total of 407,573,563 sequenced bases were obtained, with a read N50 of 6082 and a median quality score of 10.6. Reads shorter than 500 bp were removed using filtlong v0.2.1 (https://github.com/rrwick/Filtlong). The genome was then assembled using Flye v2.9^38^, producing a single circular contig. The assembly was polished using Medaka v1.6.0 (https://github.com/nanoporetech/medaka), and set to a consistent start coordinate using Circlator v1.5.5^39^. The average read coverage of the final genome was 107x. Genome quality was assessed using CheckM v1.1.2^40^. The final genome was compared with the *Eggerthella lenta* DSM 2243 reference genome obtained from NCBI RefSeq (accession GCF_000024265.1) by whole-genome alignment using mummer2circos v1.2 (https://github.com/metagenlab/mummer2circos). Nanopore reads were also aligned to the *Eggerthella lenta* DSM 2243 reference genome using minimap2 v2.24^41^. The resulting alignments were visualized using Rsamtools v2.8.0^42^ and ggplot2 v3.3.6^43^ in R v4.1.1. Variant calling versus the reference genome was performed using *medaka_haploid_variant* in Medaka v1.6.

### Gnotobiotic mouse studies

C57BL/6J mice (males ages 6–8 weeks) were obtained from the University of California, San Francisco (UCSF) Gnotobiotics core facility (gnotobiotics.ucsf.edu) and housed in Iso positive cages (Tecniplast). Mice were colonized via oral gavage with mono-cultures of *E. lenta* (10^8^–10^9^ CFUs/ml, 200 μL gavage) and colonization was confirmed via anaerobic culturing. Mice were colonized for 2 weeks with both WT control and Δ*cgr* strains. CFU counts were performed by making serial dilutions of fecal pellets from Day 0 and Day 14 and plating on BHI+ agar plates to grow at 37 °C in an anaerobic chamber. Three mice colonized with WT *E. lenta* were found to be contaminated with *Paenibacillus* and were removed from all analyses. All mouse experiments were approved by the University of California, San Francisco Institutional Animal Care and Use Committee. The mice were housed at temperatures ranging from 67 to 74 °F and humidity ranging from 30 to 70% light/dark cycle 12 h/12 h. Labdiet 5021 was used. No mice were involved in previous procedures before experiments were performed. Mice were assigned to groups to achieve similar age distribution between groups.

### Th17 skewing assay

Red blood cell (RBC) lysed mouse splenocytes from male or female C57BL/6J mice were filtered through a 40 μm filter and used for T cell isolation. T cells were isolated via Dynabeads untouched mouse CD4 isolation kit (ThermoFisher) according to kit specifications. In a 96 well plate pre-coated with anti-CD3e (Clone: 145-2C11, Catalogue: 553057, Fisher Scientific, overnight at 37 °C at 5 μg/ml), equal cell numbers were plated and were treated with bacterial CFS or media controls with a concentration of 5% volume/volume. At the same time, Th17 skewing conditions were supplied (anti-CD28 (Clone: 37.51, Catalogue: 557393, Fisher Scientific, 10 μg/ml), anti-IFNγ (Clone: XMG1.2, Catalogue: 554409, Fisher Scientific, 2 ng/ml), anti-IL-4 (Clone: 11B11, Catalogue: 5013602, Fisher Scientific, 2 ng/ml) TGFβ (Catalogue: 7666-MB-005, Fisher Scientific, 0.3 ng/ml), IL-6 (Catalogue: 575704-BL, VWR, 20 ng/ml))^44^. Bacterial CFS was harvested from 72 h stationary cultures where bacterial cells were pelleted (2500 rpm 10 min) and the supernatant was filtered through a 0.2 μm filter to exclude cells from the CFS preparation. 2 mL aliquots of CFS were taken and adjusted to 1.5× concentration using a Savant SpeedVac Plus SC110A for 24 h. Isolated CD4+ T cells were developed in Th17 skewing conditions with bacterial CFS present for 4 days at 37 °C and then re-stimulated overnight with a cell stimulation cocktail (Fisher Scientific) containing PMA and ionomycin according to the manufacturer’s instructions, then supernatants were harvested for IL-17a quantification via ELISA.

### ELISAs

To measure the levels of secreted IL-17a from the Th17 cell culture assay, the IL-17A (homodimer) Mouse Uncoated ELISA Kit (ThermoFisher) was used according to the manufacturer’s instructions. 100 μL of cell culture media was used as input. Absorbance for ELISA was measured at 450 nm, and the blank background signal was subtracted. Raw values for IL-17a ELISAs are provided as a Source Data file.

### Lamina propria lymphocyte isolation

Lamina propria lymphocytes (LPLs) were isolated with modifications of previously described methods^45–47^. Briefly, the ileum (lower 1/3 of the small intestine) and colon were splayed longitudinally with mucus removed and stored in complete RPMI (10% fetal bovine serum, 100 units per ml penicillin and streptomycin, β-mercaptoethanol, glutamax, sodium pyruvate, hydroxyethyl piperazineethanesulfonic acid (HEPES) and non-essential amino acids) on ice. Media was removed by filtering through a 100 μm filter, and remaining tissue incubated in 1× Hank’s Balanced Salt Solution (HBSS without Ca^2+^ and Mg^2+^) containing 5 mM ethylenediaminetetraacetic acid (EDTA) and 1 mM DL-Dithiothreitol (DTT) for 45 min at 37 °C on a shaker (200 rpm). Supernatant were filtered through a 100 μm filter, and remaining tissue was incubated for 45 min (colon) or 35 min (ileum) at 37 °C on a shaker in a solution containing 1× HBSS containing 5% (v/v) fetal bovine serum (GIBCO heat inactivated), 1 U/ml Dispase (Sigma), 0.5 mg/ml Collagenase VIII (Sigma), and 20 μg/ml DNaseI (Sigma). The vortexed supernatant was filtered over a 40 μm cell strainer into 1× PBS. Cells were subjected to a Percoll (VWR) gradient (40%/80% [v/v] gradient) and spun at 2000 rpm for 20 min with no brake and no acceleration. Cells at the interface were collected, washed in 1× PBS, and prepared for flow cytometry analysis as described in the next section.

### Flow cytometry

Lymphocytes were isolated from the colonic and ileum lamina propria as described above. For intracellular staining, cells were stimulated with a cell stimulation cocktail (Fisher Scientific) containing PMA and ionomycin according to the manufacturer’s instructions, and Golgi plug was added (1 μL/sample) (BD Bioscience) and incubated 4–6 hours at 37 °C. Surface staining for lymphocytes was done in staining buffer (1× HBSS (Corning) supplemented with 10 mM HEPES (Fisher Scientific), 2 mM EDTA (Invitrogen), and 0.5% (v/v) fetal bovine serum (GIBCO heat inactivated)) for 20 min at 4 °C. Cells were then washed twice in staining buffer. The following antibodies were used for extracellular staining: anti-CD3 (Clone: 17A2, Catalogue: 11-0032-82, Fisher Scientific, 0.2:100), anti-CD4 (Clone: GK1.5, Catalogue: BDB563331, Biolegend, 0.2:100), and live/dead staining was performed using LIVE/DEAD Fixable Dead Cell Stain Kit (Life Technologies, dilution 1:100). Cells were then fixed/permeabilized in 100 μL fixation and permeabilization (Perm) buffer (BD Bioscience). Cells were washed twice in Perm/Wash buffer (BD Bioscience) and then stained for intracellular cytokine with anti-IL17a (Clone: ebio17B7, Catalogue: 25-7177-82, Fisher Scientific, 5:100). Cells were washed twice in Perm/Wash buffer and then placed in staining buffer for flow cytometry analysis. Gating cell populations was done using isotype and single stain controls. Gating strategies are outlined in Supplementary Fig. 8f. The flow cytometry data were collected with a BD LSR Fortessa and analyzed with FlowJo software (version 10.7.1).

### Quantification and statistical analysis

Unless otherwise specified, statistical analysis was carried out using Prism 9 (GraphPad Software). Individual datapoints have been shown where possible but are otherwise represented as the mean ± standard deviation unless otherwise stated.

### Reporting summary

Further information on research design is available in the Nature Research Reporting Summary linked to this article.

## Supplementary information

Supplementary Information

Description of Additional Supplementary Files

Supplementary Dataset 1

Supplementary Dataset 2

Supplementary Dataset 3

Supplementary Dataset 4

Supplementary Dataset 5

Supplementary Dataset 6

Reporting Summary

## Data Availability

Long-read sequencing data files are available at NCBI BioProject accession PRJNA866504. The *Eggerthella lenta* DSM 2243 reference genome was obtained from NCBI RefSeq (accession GCF_000024265.1). All other data generated or analyzed during this study are included in this article and its supplementary files. Source data are provided with this paper. The plasmids used in this study are available on Addgene (Emily Balskus Lab Materials). Further information and requests for resources and reagents should be directed to and will be fulfilled by the Lead Contact, Emily P. Balskus (balskus@chemistry.harvard.edu). Source data are provided with this paper.

## Source data

Source Data
