# Supplementary material for: Genetic manipulation of the human gut bacterium Eggerthella lenta reveals a widespread family of transcriptional regulators
Source: Nat Commun. 2022 Dec 9;13:7624. doi: 10.1038/s41467-022-33576-3 (PMC9734109; doi:10.1038/s41467-022-33576-3)
Supplement: Supplementary file 1 — Supplementary Information [file 41467_2022_33576_MOESM1_ESM.pdf]

Supplementary Information

Genetic manipulation of the human gut bacterium *Eggerthella lenta* reveals a widespread family of transcriptional regulators

Authors: Xueyang Dong[1], Ben G. H. Guthrie[2], Margaret Alexander[2], Cecilia Noecker[2], Lorenzo Ramirez[2], Nathaniel R. Glasser[1], Peter J. Turnbaugh[2,3], Emily P. Balskus[1,4*]

[1]Department of Chemistry and Chemical Biology, Harvard University, Cambridge, MA 02138, USA

[2]Department of Microbiology & Immunology, University of California San Francisco, San Francisco, CA 94143, USA

[3]Chan Zuckerberg Biohub, San Francisco, CA 94158, USA

[4]Howard Hughes Medical Institute, Harvard University, Cambridge, MA 02138, USA

*Correspondence: balskus@chemistry.harvard.edu

This file includes:

Supplementary Figures 1–8

Supplementary Tables 1

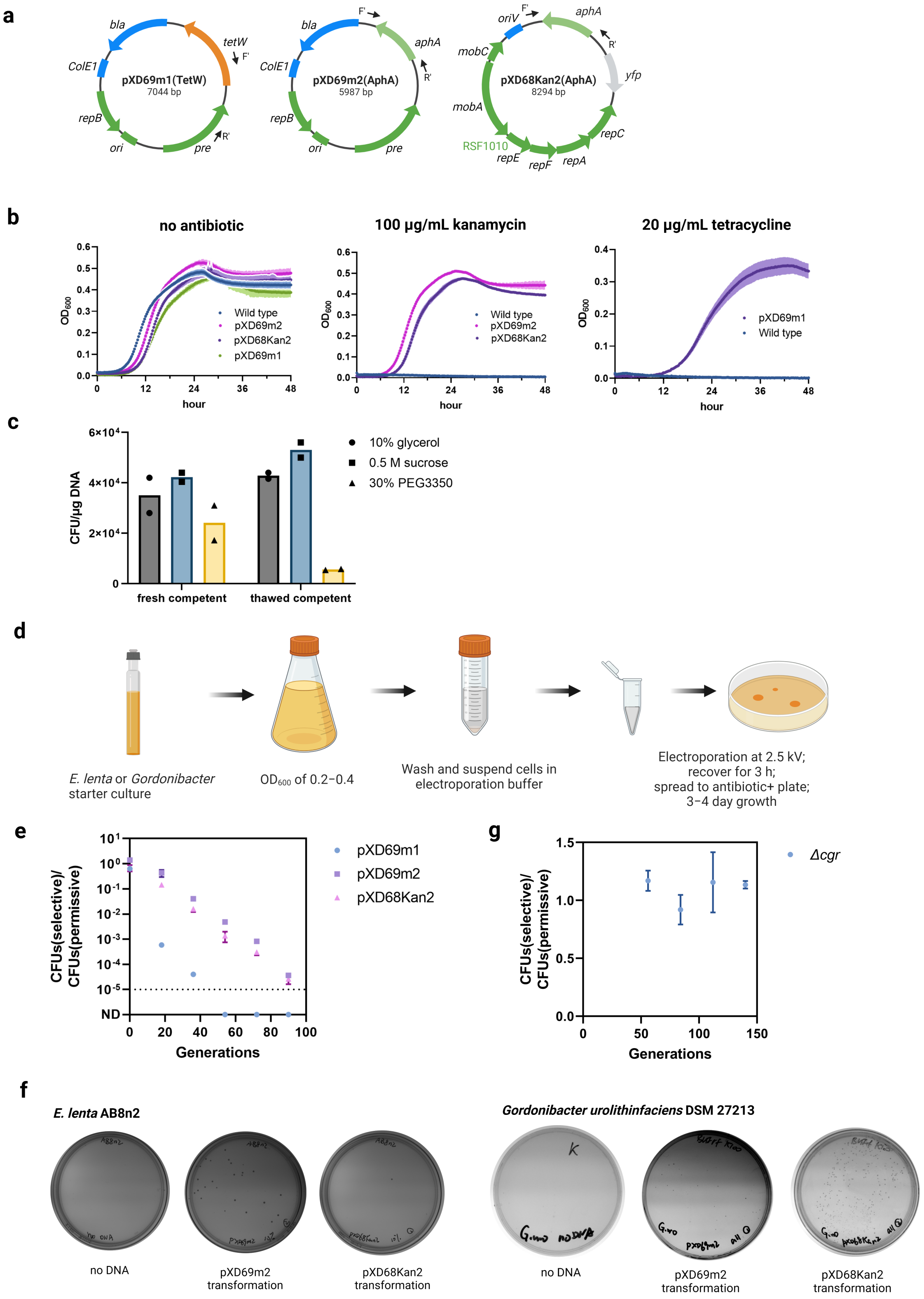
a
bla
tetW
F'
ColE1
pXD69m1(TetW)
7044 bp
repB
ori
pre
R'
bla
F'
aphA
ColE1
pXD69m2(AphA)
5987 bp
R'
repB
ori
pre
oriV
F'
aphA
mobC
R'
yfp
pXD68Kan2(AphA)
8294 bp
mobA
repC
RSF1010
repE
repF
repA
b
no antibiotic
100 μg/mL kanamycin
20 μg/mL tetracycline
OD600
hour
Wild type
pXD69m2
pXD68Kan2
pXD69m1
c
CFU/μg DNA
6×10⁴
4×10⁴
2×10⁴
fresh competent
thawed competent
10% glycerol
0.5 M sucrose
30% PEG3350
d
E. lenta or Gordonibacter starter culture
OD600 of 0.2–0.4
Wash and suspend cells in electroporation buffer
Electroporation at 2.5 kV; recover for 3 h; spread to antibiotic+ plate; 3–4 day growth
e
CFUs(selective)/ CFUs(permissive)
Generations
ND
pXD69m1
pXD69m2
pXD68Kan2
g
CFUs(selective)/ CFUs(permissive)
Generations
Δcgr
f
E. lenta AB8n2
Gordonibacter urolithinfaciens DSM 27213
no DNA
pXD69m2 transformation
pXD68Kan2 transformation
no DNA
pXD69m2 transformation
pXD68Kan2 transformation

Supplementary Fig. 1| Transformation of human gut Coriobacteriia related to Fig. 1. a, Arrows indicating regions amplified on individual plasmid to confirm plasmid presence within *E. lenta* and *Gordonibacter* transformants. **b**, Growth of *E. lenta* DSM 2243 pXD69m1, pXD69m2, and pXD68Kan2 transformants and WT strain in liquid BHIrf medium or medium supplemented with 100 μg/mL kanamycin or 20 μg/mL tetracycline. **c**, Additional buffers, 0.5 M sucrose solution or 30% PEG3350 solution, could be used to prepare *E. lenta* DSM 2243 electrocompetent cells. Electrocompetent *E. lenta* cells made with 10% glycerol or 0.5 M sucrose tolerated freezing. We thus used 10% glycerol to prepare *E. lenta* DSM 2243 electrocompetent cells and stored competent cells at –80 °C for further routine transformation unless otherwise noted. **d**, Workflow of preparing *E. lenta* and *Gordonibacter* species electrocompetent cells. **e**, Plasmid maintenance of pXD69m1, pXD69m2 and pXD68Kan2 in *E. lenta* DSM 2243. ND: colonies not detected in the selective medium. **f**, Transformation of additional *E. lenta* and *Gordonibacter* species strains. Electrocompetent cells for *E. lenta* 28B, A2, AB8n2, AB12n2, CC8/2 BHI2 and Valencia strains, *Gordonibacter pamelaeae* 3C, *Gordonibacter sp.* 28C and *Gordonibacter urolithinfaciens* DSM 27213 were prepared following the same procedures for *E. lenta* DSM 2243. We found plasmids can be transformed into *E. lenta* AB8n2, *Gordonibacter urolithinfaciens* DSM 27213 and *Gordonibacter sp.* 28C, but not others, using our electroporation conditions. **g**, Plasmid maintenance of *cgr*-editing plasmid in *E. lenta* DSM 2243 Δ*cgr*. Results represented as mean with n = 2 biological replicates for **c**. Results represented as mean ± standard deviation (SD) with n = 3 biological replicates for **b**, **e** and **g**. Source data are provided as a Source Data file.

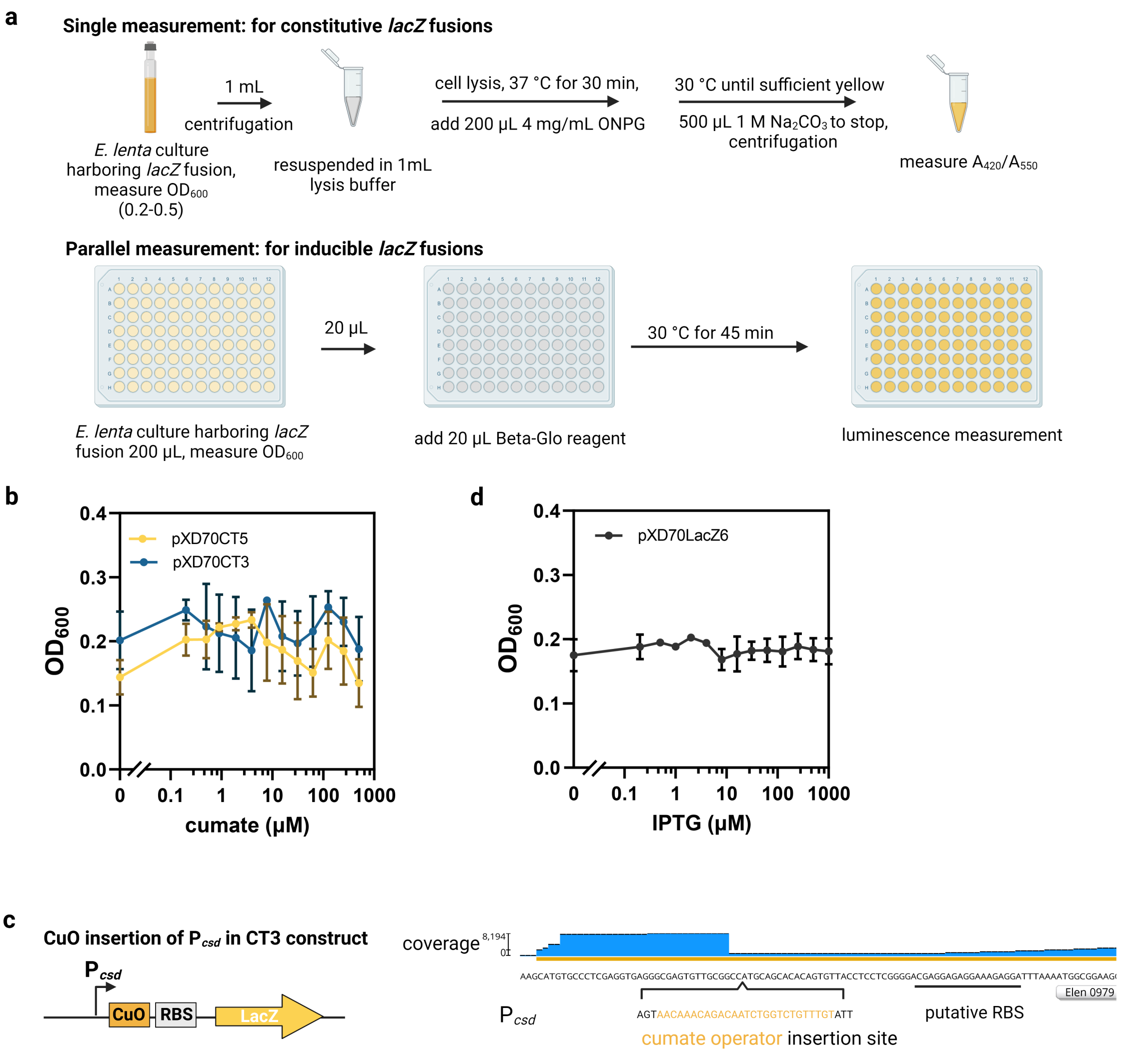

Supplementary Fig. 2| Construction and characterization of inducible expression systems using *lacZ* reporter related to Fig. 2. a, Workflow of *lacZ* assays to measure β-Gal activity of *E. lenta* cultures harboring different *lacZ* fusions. **b**, Growth of *E. lenta* DSM 2243 cultures harboring pXD70CT3 or pXD70CT5 in the presence of different concentrations of cumate overnight at 37 ˚C. Final OD_{600} represented as mean ± SD with n = 3 biological replicates. **c**, To identify an optimal site for CuO insertion on P_{csd}, transcriptomic reads from previous *E. lenta* RNA-seq experiments[1] were mapped to the P_{csd} region. A cumate operator was inserted near a site where we found an abrupt decrease of RNA read coverage, as we speculated that binding of CymR at this site may establish control of gene expression. **d**, Growth of *E. lenta* DSM 2243 cultures harboring pXD70LacZ6 in the presence of different concentrations of IPTG overnight at 37 ˚C. Final OD_{600} represented as mean ± SD with n = 3 biological replicates for **b** and **d**. Source data are provided as a Source Data file.

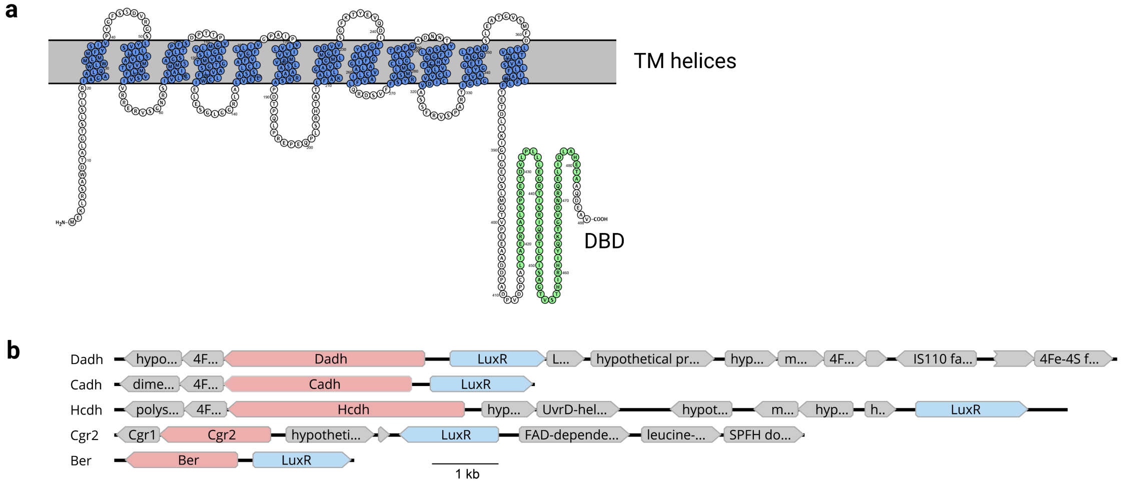

Supplementary Fig. 3| Domain architecture of DadR and genomic arrangement of 12-transmembrane helix LuxRs in *E. lenta* DSM 2243 genome. a, DadR is predicted to possess an N-terminal 12-transmembrane helix domain (blue) and a C-terminal helix-turn-helix DNA-binding domain (green) by TMHMM[2]. Membrane topology model was generated by Protter[3]. **b**, Predicted 12-TM LuxRs (blue) are located near genes encoding *E. lenta* metabolic enzymes Dadh, Cadh, Hcdh, Cgr2, and Ber (red).

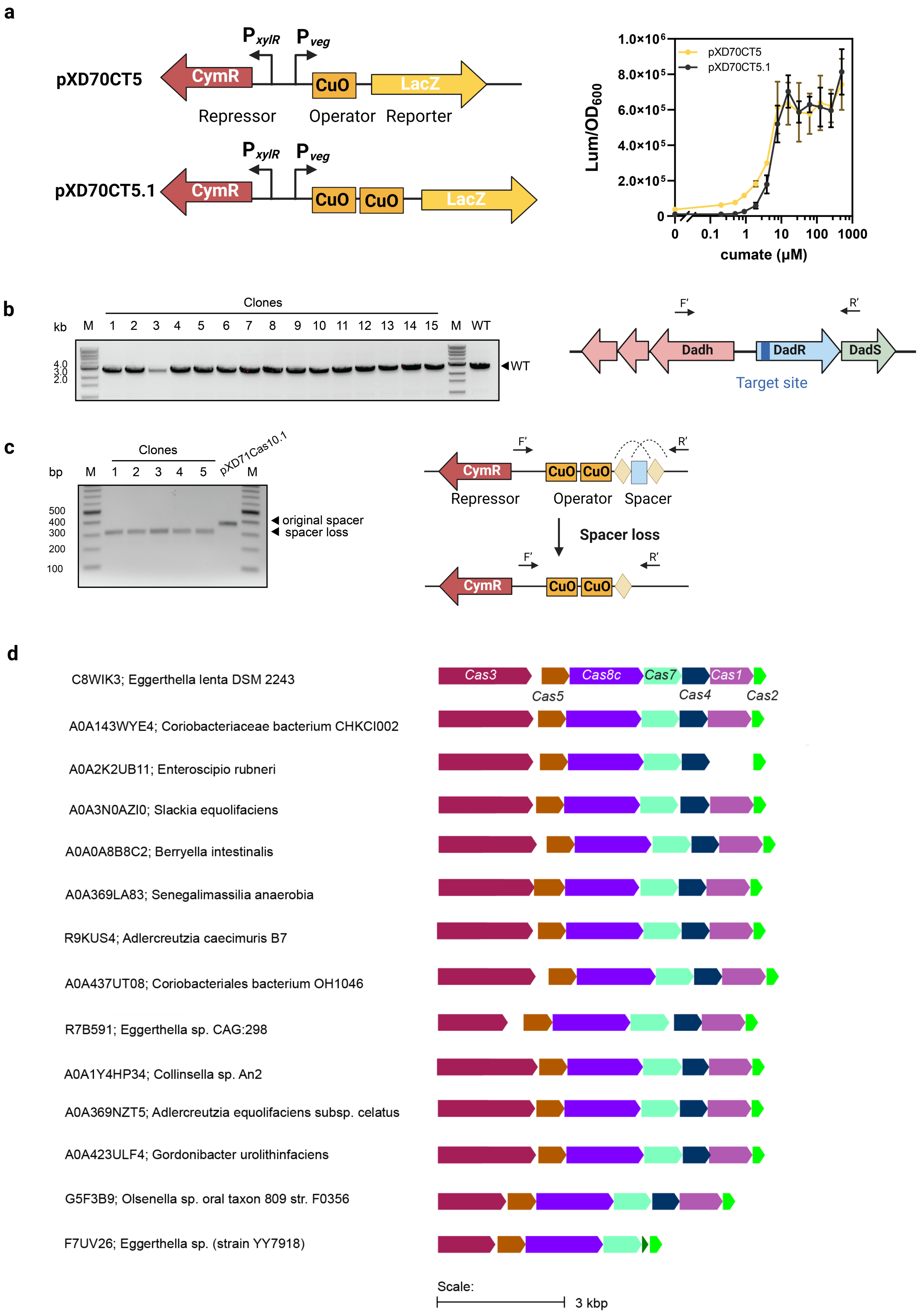

a
pXD70CT5
pXD70CT5.1
CymR
CuO
LacZ
Repressor
Operator
Reporter
Lum/OD600
cumate (μM)
1.0×10⁶
8.0×10⁵
6.0×10⁵
4.0×10⁵
2.0×10⁵
0
0.1
1
10
100
1000
b
Clones
kb
M
1
2
3
4
5
6
7
8
9
10
11
12
13
14
15
WT
4.0
3.0
2.0
Dadh
DadR
DadS
Target site
F′
R′
c
bp
pXD71Cas10.1
500
400
300
200
100
original spacer
spacer loss
Spacer
Spacer loss
d
C8WIK3; Eggerthella lenta DSM 2243
A0A143WYE4; Coriobacteriaceae bacterium CHKCI002
A0A2K2UB11; Enteroscipio rubneri
A0A3N0AZI0; Slackia equolifaciens
A0A0A8B8C2; Berryella intestinalis
A0A369LA83; Senegalimassilia anaerobia
R9KUS4; Adlercreutzia caecimuris B7
A0A437UT08; Coriobacteriales bacterium OH1046
R7B591; Eggerthella sp. CAG:298
A0A1Y4HP34; Collinsella sp. An2
A0A369NZT5; Adlercreutzia equolifaciens subsp. celatus
A0A423ULF4; Gordonibacter urolithinfaciens
G5F3B9; Olsenella sp. oral taxon 809 str. F0356
F7UV26; Eggerthella sp. (strain YY7918)
Cas3
Cas5
Cas8c
Cas7
Cas4
Cas1
Cas2
Scale:
3 kbp

Supplementary Fig. 4| Harnessing endogenous Type I-C CRISPR-Cas system for *E. lenta* genomic engineering, related to Fig. 5. a, Adding an additional CuO tightened the expression control of cumate-inducible construct. *LacZ* assays revealed that pXD70CT5.1 construct showed lower *lacZ* expression levels than pXD70CT5 in the absence of cumate and at low cumate concentrations. Results represented as mean ± SD with n = 3 biological replicates. **b**, PCR screening was performed to probe potential *dadR* deletion in colonies formed after spreading initial pXD71Cas10.1 transformant cultures onto a cumate-containing agar plate, and revealed all the tested colonies lacked *dadR* deletion. *E. lenta* DSM 2243 WT gDNA was used as control. Primers flanking the target site were used. **c**, PCR screening revealed spacer loss within colonies formed after spreading pXD71Cas10.1 transformant cultures onto a cumate-containing agar plate, which was confirmed by Sanger sequencing. pXD71Cas10.1 plasmid was used as control. **d**, Presence of Type I-C CRISPR-Cas systems in Coriobacteriia. The amino acid sequence of *E. lenta* DSM 2243 Cas3 (C8WIK3) was blast searched against Coriobacteriia genomes in UniProt database, and genomic contexts of the hits were retrieved and visualized using EFI-GNT tool[4] and manually curated according to the presence of other typical Cas proteins within genome neighborhoods. For each diagram, the accession of the Cas3 homolog, species identity and the CRISPR-Cas locus were displayed. Experiments shown in panels **b** and **c** were performed once on randomly selected colonies. Source data are provided as a Source Data file.

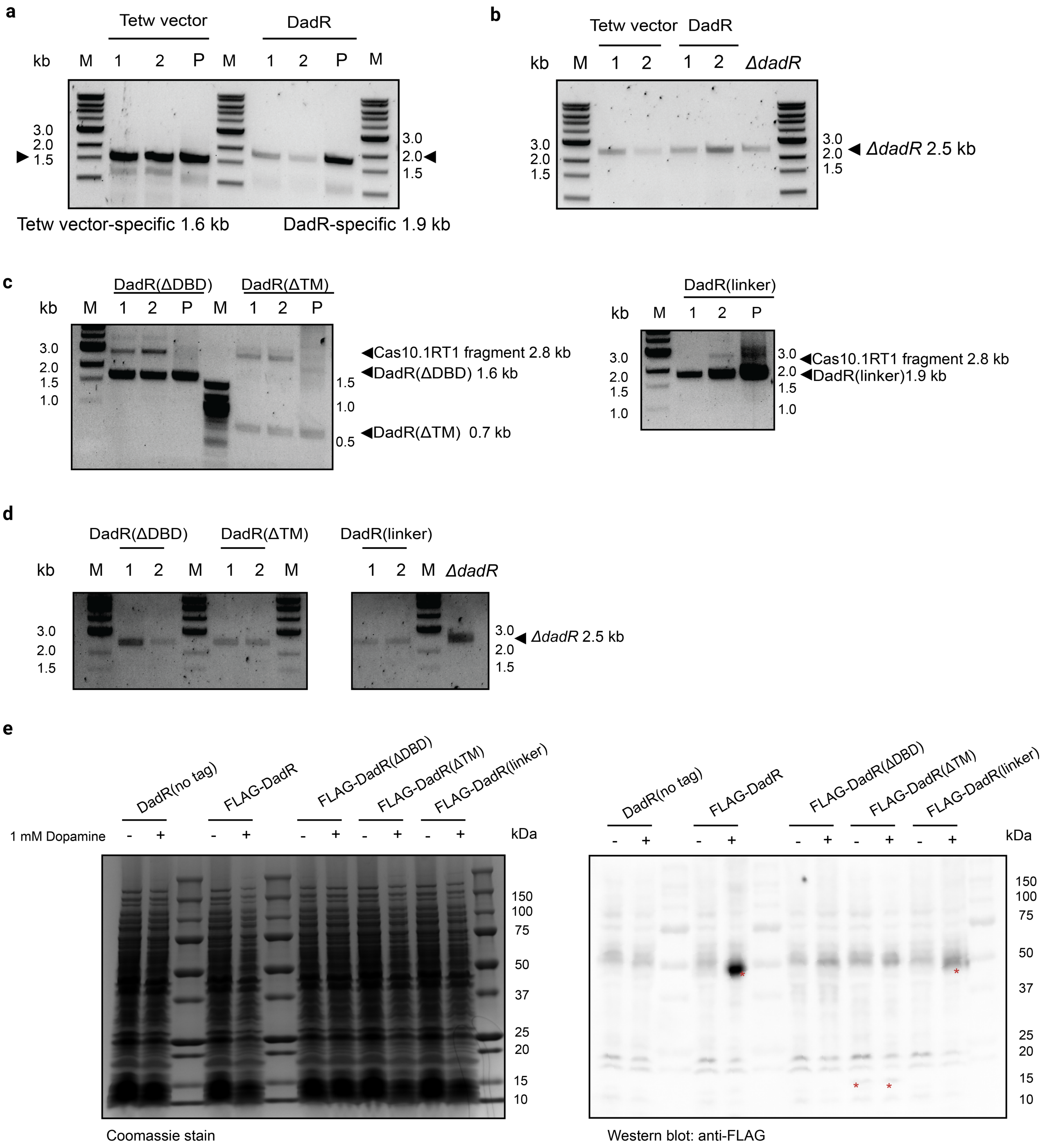

Supplementary Fig. 5| Confirmation of *dadR* gene complementation and expression related to Fig. 6. a, PCR screening confirmed the presence of tetracycline-resistance plasmid pXD69m1(TetW) vector and pXD70Tet(DadR) within the complemented strains Δ*dadR*/vector and Δ*dadR*/DadR, respectively. Plasmids (P) were used as controls. **b**, PCR screening confirmed the *dadR* deletion background of the Δ*dadR*/vector and Δ*dadR*/DadR strains. The gDNA of Δ*dadR* strain was used as a control. **c**, PCR screening confirmed the presence of the plasmid encoding truncated DadR mutant DadR(ΔDBD), DadR(ΔTM) or linker shuffled mutant DadR(linker) within the corresponding

complemented strains. **d**, PCR screening confirmed the *dadR* deletion background of the strains complemented with plasmid encoding DadR mutants. **e**, Western blot to measure the expression of N-terminal FLAG-tagged DadR and DadR mutants. *E. lenta* Δ*dadR* strains harboring various N-terminal FLAG-tagged DadR constructs were inoculated 1:20 in fresh BHIrf medium with 20 μg/mL tetracycline and were either incubated with vehicle (-) or 1 mM dopamine (+) at 37 °C for 40 h before harvesting for measuring protein expression. Molecular weight of each of the N-FLAG-tagged constructs: FLAG-DadR 56 kDa, FLAG-DadR(ΔDBD) 48 kDa, FLAG-DadR(ΔTM) 15 kDa, FLAG-DadR(linker) 56 kDa. Asterisks indicate the detected signals for individual constructs. The unspecific signals close to the expected location of FLAG-DadR(ΔDBD) prevented a steady readout of signals from FLAG-DadR(ΔDBD). Experiments shown in panels **a**–**d** were performed once on randomly selected colonies. For the experiment shown in panel **e**, the Western blots for the DadR(no tag) and Flag-DadR samples were performed twice with similar results, and Western blots for the FLAG-DadR(ΔDBD), FLAG-DadR(ΔTM) and FLAG-DadR(linker) samples were performed once. Source data are provided as a Source Data file.

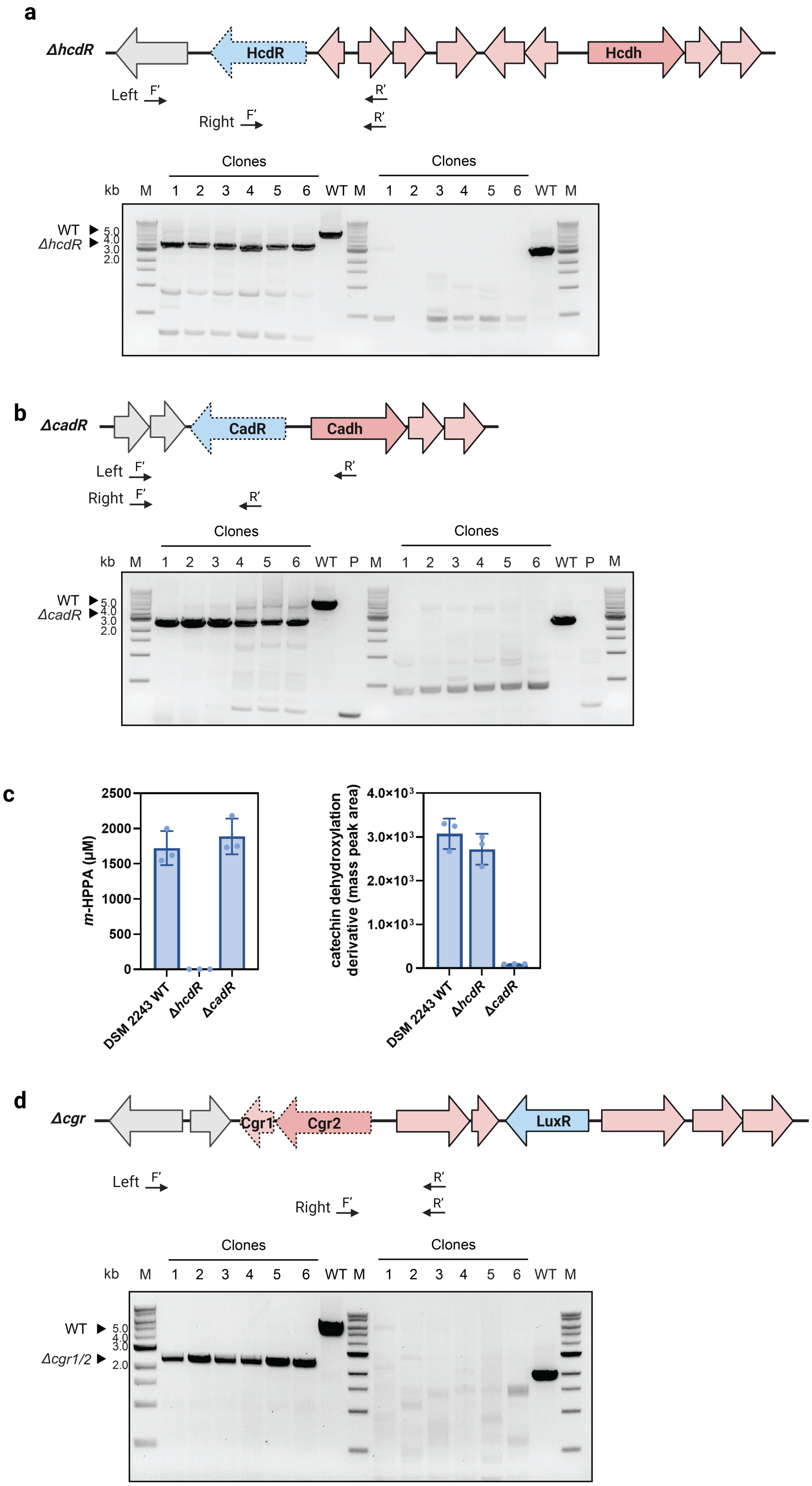
a
ΔhcdR
HcdR
Hcdh
Left F′
R′
Right F′
R′
Clones
Clones
kb M 1 2 3 4 5 6 WT M 1 2 3 4 5 6 WT M
WT
ΔhcdR
5.0
4.0
3.0
2.0
b
ΔcadR
CadR
Cadh
Left F′
R′
Right F′
R′
Clones
Clones
kb M 1 2 3 4 5 6 WT P M 1 2 3 4 5 6 WT P M
WT
ΔcadR
5.0
4.0
3.0
2.0
c
m-HPPA (μM)
2500
2000
1500
1000
500
0
DSM 2243 WT
ΔhcdR
ΔcadR
catechin dehydroxylation derivative (mass peak area)
4.0×10³
3.0×10³
2.0×10³
1.0×10³
0
DSM 2243 WT
ΔhcdR
ΔcadR
d
Δcgr
Cgr1
Cgr2
LuxR
Left F′
R′
Right F′
R′
Clones
Clones
kb M 1 2 3 4 5 6 WT M 1 2 3 4 5 6 WT M
WT
Δcgr1/2
5.0
4.0
3.0
2.0

Supplementary Fig. 6| Confirmation of additional gene deletion mutants. a,**b**,**d**, PCR screening performed to probe deletion of *E. lenta* DSM 2243 *hcdR*, *cadR*, and *cgr1*/*cgr2*. *E. lenta* DSM 2243 WT gDNA was used as control template. For *cadR* deletion, crRNA plasmid (P) was also used as control template. Two sets of primers were used for confirming each gene deletion. Left: two primers are flanking the deletion region; Right: one primer was located within the deleted region and one primer was located outside of the deletion region. The lower faint bands may be PCR amplification artifacts. **c**, LC-MS/MS to quantify the production of hydrocaffeic acid dehydroxylation metabolite *m*-HPPA and (+)-catechin dehydroxylation product after incubation with corresponding *E. lenta* cultures for 48 h. Data represented as mean ± SD with n = 3 biological replicates. Experiments shown in panels **a**, **b** and **d** were performed once on randomly selected colonies**.** Source data are provided as a Source Data file.

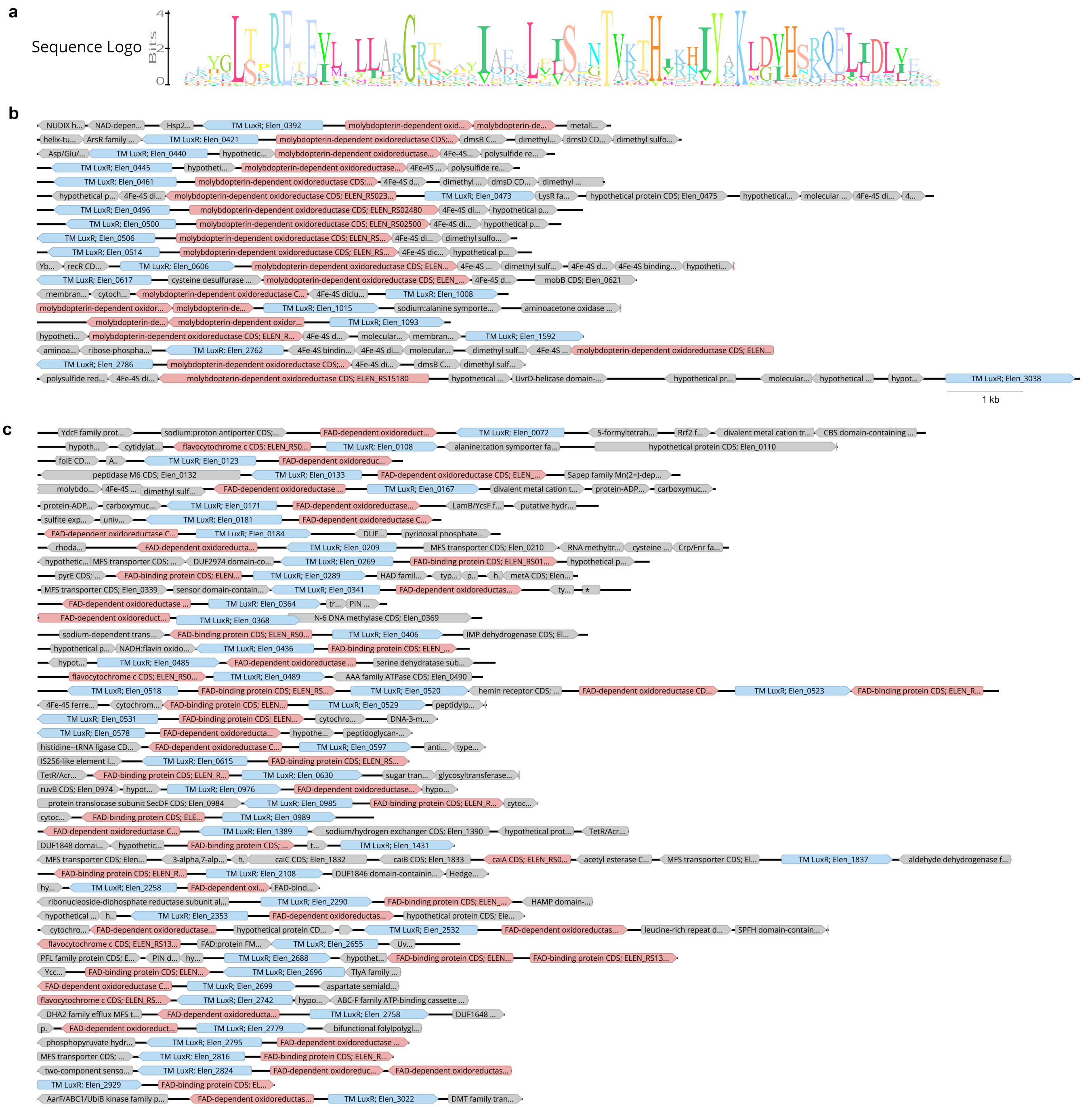

Supplementary Fig. 7| *E. lenta* 10–12-TM LuxRs share significant homology within the C-terminal DNA-binding domain and are co-localized with molybdenum- and flavin-dependent enzymes. a, Sequence alignment was performed for the 74 TM LuxR regulators in *E. lenta* DSM 2243 that are predicted to possess 10, 11 or 12 N-terminal transmembrane helices, using MUSCLE 3.8.425 in Geneious Prime 2021.2.2. The sequence logo highlights the consensus residues within the C-terminal DNA-binding domain. **b**, 19 of the *E. lenta* TM LuxRs (blue) are encoded close to molybdenum-dependent enzymes (red). **c**, 50 of the *E. lenta* TM LuxRs (blue) are encoded in proximity to flavin-dependent enzymes (red).

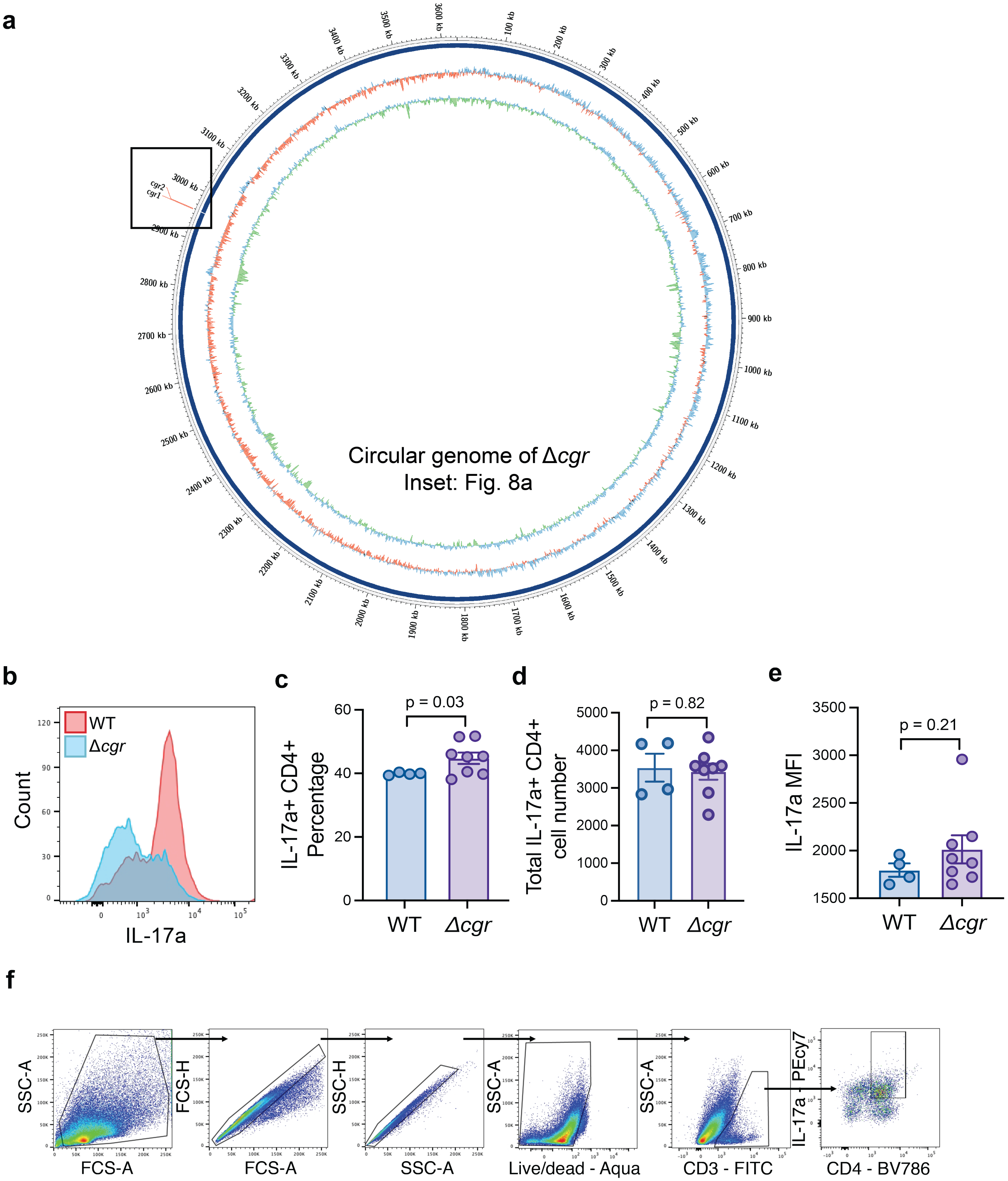
a
Circular genome of Δcgr
Inset: Fig. 8a
b
WT
Δcgr
Count
IL-17a
c
IL-17a+ CD4+
Percentage
p = 0.03
WT
Δcgr
d
Total IL-17a+ CD4+
cell number
p = 0.82
WT
Δcgr
e
IL-17a MFI
p = 0.21
WT
Δcgr
f
SSC-A
FCS-A
FCS-H
FCS-A
SSC-H
SSC-A
SSC-A
Live/dead - Aqua
SSC-A
CD3 - FITC
IL-17a - PEcy7
CD4 - BV786

Supplementary Fig. 8| Experiments related to Figure 8. a, Circos plot showing Flye assembly (blue) of the *Δcgr* genome aligned to the reference *E. lenta* DSM 2243 genome GCF_000024265.1_ASM2426v1 (figure generated with mummer2circos). **b–d**, Germ-free C57BL/6J male mice ages 6–8 weeks were separated into groups and gavaged with WT (n=4) and *Δcgr* (n=8) *E. lenta*. Bacteria were allowed to colonize for 2 weeks before the lamina propria was harvested. **b**, Representative fluorescence histograms of colonic IL-17a levels. **c**, Percentage of ileal IL-17a$^+$ CD4$^+$ cells within the live CD3$^+$ gate. **d**, Total numbers of ileal IL-17a$^+$ CD4$^+$ cells within the live CD3$^+$ gate. **e**, Mean fluorescence intensity of ileal IL-17a. **f**, Gating strategy for flow cytometry of Th17 cells. All *p*-values are displayed and were calculated with one-way ANOVA tests with Tukey's multiple correction or Welch's t tests for two-way comparisons. Data represented as mean ± SD for **c**, **d**, and **e**. Panels **b**–**e** show representative data from the first of two independent experiments. Source data are provided as a Source Data file.

Supplementary Table 1| Whole genome sequencing for the Δ*cgr* strain in comparison with other reported genomes of *E. lenta* DSM 2243.

Gene annotations are based on NCBI/Prodigal for the reference genome, and prokka v1.13.3 for DSM 2243 (UCSF) and Δ*cgr*.

Genome name	*Eggerthella lenta* DSM 2243	*Eggerthella lenta* DSM 2243 (UCSF)	*Eggerthella lenta* DSM 2243 Δ*cgr*
**Source/reporting study**	NCBI RefSeq GCF_000024265.1	(Bisanz et al. 2020)[5]	This study
**Genome size (bp)**	3632260	3596824	3641600
**Number of contigs**	1	69	1
**N50**	3632260	130831	3641600
**Coverage**	10.2x Sanger; 25.3x pyrosequence	1210.4x Illumina	107.1x Oxford Nanopore MinIon
**Number of protein-coding genes**	3062	3091	3151
**Number of tRNA genes**	49	52	52
**Number of rRNA genes**	9	3	9
**GC content**	64.20%	64.19%	64.19%
**Completeness**	100%	100%	98.80%
**Contamination**	0%	0%	0%
**SNPs vs DSM 2243 reference**	N/A	24	120
**Indels vs DSM 2243 reference**	N/A	17	106

Supplementary References

1 Rekdal, V. M., Bess, E. N., Bisanz, J. E., Turnbaugh, P. J. & Balskus, E. P. Discovery and inhibition of an interspecies gut bacterial pathway for Levodopa metabolism. *Science* **364**, eaau6323 (2019).

2 Krogh, A., Larsson, B., von Heijne, G. & Sonnhammer, E. L. Predicting transmembrane protein topology with a hidden Markov model: application to complete genomes. *J. Mol. Biol.* **305**, 567-580 (2001).

3 Omasits, U., Ahrens, C. H., Muller, S. & Wollscheid, B. Protter: interactive protein feature visualization and integration with experimental proteomic data. *Bioinformatics* **30**, 884-886 (2014).

4 Zallot, R., Oberg, N. & Gerlt, J. A. The EFI Web Resource for Genomic Enzymology Tools: Leveraging Protein, Genome, and Metagenome Databases to Discover Novel Enzymes and Metabolic Pathways. *Biochemistry* **58**, 4169-4182 (2019).

5 Bisanz, J. E. *et al.* A Genomic Toolkit for the Mechanistic Dissection of Intractable Human Gut Bacteria. *Cell Host Microbe* **27**, 1001-1013 e1009 (2020).
