# Supplementary material for: Genetic manipulation of the human gut bacterium Eggerthella lenta reveals a widespread family of transcriptional regulators
Source: Nat Commun. 2022 Dec 9;13:7624. doi: 10.1038/s41467-022-33576-3 (PMC9734109; doi:10.1038/s41467-022-33576-3)
Supplement: Supplementary file 2 — Description of Additional Supplementary Files [file 41467_2022_33576_MOESM2_ESM.pdf]

Description of Additional Supplementary Files

File Name: Supplementary Data 1

Description: List of *E. lenta* DSM 2243 10–12 transmembrane helix LuxRs

File Name: Supplementary Data 2

Description: MUSCLE alignment of *E. lenta* 10–12 transmembrane helix LuxRs.

File Name: Supplementary Data 3

Description: List of Coriobacteriia 10–12 transmembrane helix LuxRs.

File Name: Supplementary Data 4

Description: List of proteins containing N-terminal 10–12 transmembrane domain and C-terminal LuxR-type helix-turn-helix DNA-binding domain (PF00196) obtained from UniProt.

File Name: Supplementary Data 5

Description: Oligonucleotide sequences used in the study.

File Name: Supplementary Data 6

Description: Nucleotide sequences of genetic parts used in the study.
