# Supplementary material for: Genetic manipulation of the human gut bacterium Eggerthella lenta reveals a widespread family of transcriptional regulators
Source: Nat Commun. 2022 Dec 9;13:7624. doi: 10.1038/s41467-022-33576-3 (PMC9734109; doi:10.1038/s41467-022-33576-3)
Supplement: Supplementary file 9 — Reporting Summary [file 41467_2022_33576_MOESM9_ESM.pdf]

nature portfolio

Corresponding author(s): Emily P. Balskus

Last updated by author(s): Sep 7, 2022

Reporting Summary

Nature Portfolio wishes to improve the reproducibility of the work that we publish. This form provides structure for consistency and transparency in reporting. For further information on Nature Portfolio policies, see our Editorial Policies and the Editorial Policy Checklist.

Statistics

For all statistical analyses, confirm that the following items are present in the figure legend, table legend, main text, or Methods section.

n/a	Confirmed	
☐	☒	The exact sample size (*n*) for each experimental group/condition, given as a discrete number and unit of measurement
☐	☒	A statement on whether measurements were taken from distinct samples or whether the same sample was measured repeatedly
☐	☒	The statistical test(s) used AND whether they are one- or two-sided<br>*Only common tests should be described solely by name; describe more complex techniques in the Methods section.*
☒	☐	A description of all covariates tested
☒	☐	A description of any assumptions or corrections, such as tests of normality and adjustment for multiple comparisons
☐	☒	A full description of the statistical parameters including central tendency (e.g. means) or other basic estimates (e.g. regression coefficient) AND variation (e.g. standard deviation) or associated estimates of uncertainty (e.g. confidence intervals)
☐	☒	For null hypothesis testing, the test statistic (e.g. *F*, *t*, *r*) with confidence intervals, effect sizes, degrees of freedom and *P* value noted<br>*Give P values as exact values whenever suitable.*
☒	☐	For Bayesian analysis, information on the choice of priors and Markov chain Monte Carlo settings
☒	☐	For hierarchical and complex designs, identification of the appropriate level for tests and full reporting of outcomes
☒	☐	Estimates of effect sizes (e.g. Cohen's *d*, Pearson's *r*), indicating how they were calculated

Our web collection on statistics for biologists contains articles on many of the points above.

Software and code

Policy information about availability of computer code

Data collection	No software was used for data collection.
Data analysis	Statistical analysis: Graphpad Prism 9<br>Sequence alignment: MUSCLE 3.8.425 in Geneious Prime 2021.2.2<br>Transmembrane helix prediction: TMHMM - 2.0<br>Nanopore data processing: MinKnow core v5.0.0 with Guppy v6.0.7<br>Long read QC: Filtlong v0.2.1<br>Long read assembly: Flye v2.9<br>Assembly polishing and variant calling: Medaka v1.6.0<br>Genome curation: Circlator v1.5.5<br>Genome QC: CheckM v1.1.2<br>Whole genome alignment: mummer2circos v1.2<br>Long read alignment: minimap2 v2.24<br>Alignment visualization: R v4.4.1, Rsamtools v2.8.0, and ggplot2 v3.3.6

For manuscripts utilizing custom algorithms or software that are central to the research but not yet described in published literature, software must be made available to editors and reviewers. We strongly encourage code deposition in a community repository (e.g. GitHub). See the Nature Portfolio guidelines for submitting code & software for further information.

Data

Policy information about availability of data

All manuscripts must include a data availability statement. This statement should provide the following information, where applicable:

- Accession codes, unique identifiers, or web links for publicly available datasets
- A description of any restrictions on data availability
- For clinical datasets or third party data, please ensure that the statement adheres to our policy

Long-read sequencing data files are available at NCBI BioProject accession PRJNA866504. The Eggerthella lenta DSM 2243 reference genome was obtained from NCBI RefSeq (accession GCF_000024265.1). All other data generated or analyzed during this study are included in this article and its supplementary files. Source data are provided with this paper. The plasmids used in this study will be available on Addgene upon publication of the manuscript. Further information and requests for resources and reagents should be directed to and will be fulfilled by the Lead Contact, Emily P. Balskus (balskus@chemistry.harvard.edu).

Field-specific reporting

Please select the one below that is the best fit for your research. If you are not sure, read the appropriate sections before making your selection.

[x] Life sciences [ ] Behavioural & social sciences [ ] Ecological, evolutionary & environmental sciences

For a reference copy of the document with all sections, see nature.com/documents/nr-reporting-summary-flat.pdf

Life sciences study design

All studies must disclose on these points even when the disclosure is negative.

Sample size	Sample size was chosen as three biological replicates, matching the standard in the microbiology field [e.g., Erez and Steinberger-Levy et al. Nature 541, 488–493 (2017)]. All datapoints displayed in this study are available in the source data for others to access and analyze. Three or four biological replicates were used for statistical calculation. Individual datapoints have been shown where possible but are otherwise represented as the mean ± standard deviation unless otherwise stated. Sample size for mouse experiments and Th17 skewing was based on previous experiments (Alexander et al., Cell Host & Microbe, P17-30.E9 (2020)) investigating E. lenta-mediated activation of Th17 cells.
Data exclusions	In gnotobiotic mouse studies, three mice colonized with WT E. lenta were found to be contaminated with Paenibacillus and were removed from all analyses.
Replication	At least three replicates were used for each experiment, except that two replicates were used for results in Supplementary Fig. 1c. Replicates were performed as required, either in parallel or sequentially. All data points were plotted and are available in the source data file. Two gnotobiotic mouse experiments were performed independently. All attempts at replication were successful.
Randomization	Randomization was not formally implemented in this study, however, the choice of wells and positioning of culture tubes used in any given experiment was not pre-assigned and was therefore chosen randomly at the time of setup. For the mouse experiments, mice were assigned to groups to achieve similar age distribution between the groups.
Blinding	Blinding was not formally applied in this study. The investigators setting up the assays also analyzed the data.

Reporting for specific materials, systems and methods

We require information from authors about some types of materials, experimental systems and methods used in many studies. Here, indicate whether each material, system or method listed is relevant to your study. If you are not sure if a list item applies to your research, read the appropriate section before selecting a response.

Materials & experimental systems

n/a	Involved in the study	
[ ]	[x]	Antibodies
[x]	[ ]	Eukaryotic cell lines
[x]	[ ]	Palaeontology and archaeology
[ ]	[x]	Animals and other organisms
[x]	[ ]	Human research participants
[x]	[ ]	Clinical data
[x]	[ ]	Dual use research of concern

Methods

n/a	Involved in the study	
[x]	[ ]	ChIP-seq
[ ]	[x]	Flow cytometry
[x]	[ ]	MRI-based neuroimaging

Antibodies

Antibodies used	Anti-FLAG M2 antibody (F1804-50UG,Sigma); anti-Mouse IgG–Peroxidase antibody (A4416-.5ML, Sigma); anti-CD3 (Clone: 17A2, Catalog: 11-0032-82, Fisher Scientific, 0.2:100), anti-CD4 (Clone: GK1.5, Catalog: BDB563331 , Biolegend, 0.2:100); anti-IL17a (Clone: ebio17B7, Catalog: 25-7177-82, Fisher Scientific, 5:100). For Th17 skewing assay: anti-CD3e (Clone: 145-2C11, Catalog: 553057, Fisher Scientific, 5 μg/ml), anti-CD28 (Clone: 37.51, Catalog: 557393, Fisher Scientific, 10 μg/ml), anti-IFNγ (Clone: XMG1.2, Catalog: 554409, Fisher Scientific, 2 ng/ml), anti-IL-4 (Clone: 11B11, Catalog: 5013602, Fisher Scientific, 2 ng/ml)
Validation	Validation of the use of Anti-FLAG M2 antibody in WB has been provided by the manufacture's website. https://www.sigmaaldrich.com/US/en/product/sigma/f1804<br><br>Validation of the use of anti-Mouse IgG–Peroxidase antibody in WB has been provided by the manufacture's website. https://www.sigmaaldrich.com/US/en/product/sigma/a4416<br><br>Validation of the use of anti-CD3 has been provided by the manufacture's website. https://www.thermofisher.com/antibody/product/CD3-Antibody-clone-17A2-Monoclonal/11-0032-82<br><br>Validation of the use of anti-CD4 has been provided by the manufacture's website. https://www.fishersci.com/shop/products/anti-cd4-clone-gk1-5-bd-3/BDB563331<br><br>Validation of the use of anti-IL17a has been provided by the manufacture's website. https://www.thermofisher.com/antibody/product/IL-17A-Antibody-clone-eBio17B7-Monoclonal/25-7177-82<br><br>Validation of the use of anti-CD3e has been provided by the manufacture's website. https://www.bdbiosciences.com/en-us/products/reagents/flow-cytometry-reagents/research-reagents/single-color-antibodies-ruo/purified-na-le-hamster-anti-mouse-cd3e.553057<br><br>Validation of the use of anti-CD28 has been provided by the manufacture's website. https://www.bdbiosciences.com/en-us/products/reagents/flow-cytometry-reagents/research-reagents/single-color-antibodies-ruo/purified-hamster-anti-mouse-cd28.557393<br><br>Validation of the use of anti-IFNγ has been provided by the manufacture's website. https://www.bdbiosciences.com/en-us/products/reagents/flow-cytometry-reagents/research-reagents/single-color-antibodies-ruo/purified-rat-anti-mouse-ifn.554409<br><br>Validation of the use of anti-IL-4 has been provided by the manufacture's website. https://www.fishersci.com/shop/products/anti-m-il-4-purified-50-ug/5013602

Animals and other organisms

Policy information about studies involving animals; ARRIVE guidelines recommended for reporting animal research

Laboratory animals	C57BL/6J male mice aged 6–8 weeks were housed in Iso positive cages (Tecniplast) at temperatures ranging from 67-74 °F and humidity ranging from 30-70% light/dark cycle 12hr/12hr. Labdiet 5021 was used.
Wild animals	No wild animals were used in the study.
Field-collected samples	No field collected samples were used in the study.
Ethics oversight	All mouse experiments were approved by the University of California, San Francisco Institutional Animal Care and Use Committee.

Note that full information on the approval of the study protocol must also be provided in the manuscript.

Flow Cytometry

Plots

Confirm that:

- [x] The axis labels state the marker and fluorochrome used (e.g. CD4-FITC).
- [x] The axis scales are clearly visible. Include numbers along axes only for bottom left plot of group (a 'group' is an analysis of identical markers).
- [x] All plots are contour plots with outliers or pseudocolor plots.
- [x] A numerical value for number of cells or percentage (with statistics) is provided.

Methodology

Sample preparation	Lamina propria lymphocytes were isolated from the ileum and colon of the mice as described in the Lamina Propria Lymphocyte Isolation methods section. Cells were stimulated 4-6 hours with a cell stimulation cocktail (Fisher Scientific) containing PMA and ionomycin.
Instrument	BD LSR Fortessa
Software	FlowJo software (version 10.7.1)
Cell population abundance	No cells were sorted
Gating strategy	Gating (figure S8e) was performed as follows: lymphocytes, single cells, singles cells 2, live cells, CD3+ cells, CD4+ IL-17a+ cells. Gating cell populations was done using isotype and single stain controls.

☒ Tick this box to confirm that a figure exemplifying the gating strategy is provided in the Supplementary Information.
